# Novel oxidative routes to *N*-arylpyridoindazolium salts

**DOI:** 10.3762/bjoc.20.166

**Published:** 2024-08-07

**Authors:** Oleg A Levitskiy, Yuri K Grishin, Tatiana V Magdesieva

**Affiliations:** 1 Chemistry Department, Lomonosov Moscow State University, Leninskie Gory, 1/3, Moscow, 119234, Russia

**Keywords:** anodic oxidation, diarylamines, electrochemical cyclization, pyridoindazolium salts, reversible ring closure

## Abstract

A novel facile approach to *N*-arylpyridoindazolium salts is proposed, based on direct oxidation of the *ortho*-pyridine substituted diarylamines, either using bis(trifluoroacetoxy)iodobenzene as an oxidant, or electrochemically, via potentiostatic oxidation. Electrochemical synthesis occurs under mild conditions; no chemical reagents are required except electric current. Both approaches can be considered as a late-stage functionalization; easily available *ortho*-pyridyl-substituted diarylamines are used as the precursors.

## Introduction

Aromatic polyfused N-heterocycles are of interest as a popular structural motif of many biologically active alkaloids and other molecules of therapeutic interest [1–4]. Polycyclic N-heteroaromatics also often exhibit intercalating properties [5–9].

**Scheme 1:**
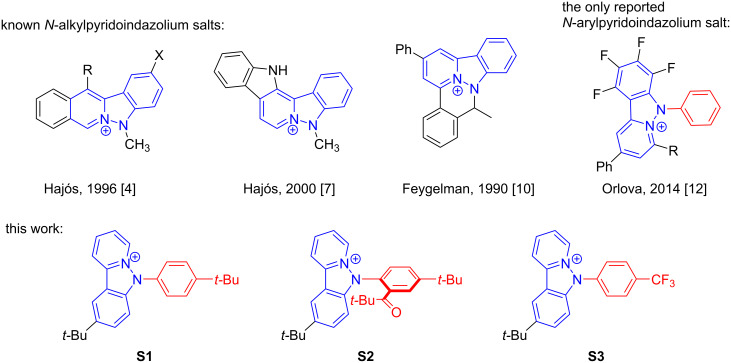
Pyridoindazolium salts known to date and obtained in the present work.

Pyridoindazolium salts can be considered as one of the typical representatives of polyfused N-heteroaromatics. However, this type of compounds is relatively rare. To the best of our knowledge, only three *N*-alkylpyridoindazolium salts [4,7,10–11] and the only *N*-aryl derivative [12] have been reported till now (Scheme 1). Meanwhile, these new ring systems are suitable for biological investigations, they exhibit significant bioactivity [4] and can be used as intercalating agents [7].

The very limited scope of these practically useful compounds is mainly attributed to the lack of convenient synthetic approaches to them. Thus, the only example of *N*-arylpyridoindazolium salts was obtained via intramolecular cyclization of perfluorinated phenylpyrilium salts using arylhydrazine [12]; the process is based on the fluorine nucleophilic substitution thus limiting its applicability to a wider range of substrates.

Pyridyl-substituted diarylamines may be considered as the possible precursors for *N*-arylpyridoindazolium salts. The oxidative behavior of substituted diarylamines is known to be very diverse and strongly influenced by the substituents in the phenyl rings as well as by the type of the oxidant. Diarylamines can serve as precursors for a wide variety of practically useful compounds such as diarylnitroxides [13–19], *N*,*N*-diarylbenzidines [20–21], *N*,*N*-diaryldihydrophenazines [20–21] and some others. Therefore, the selectivity issue is of primary importance. The guidelines for prediction of the dominant reaction path in the competing oxidative transformations of variously substituted diphenylamines yielding *N*,*N*-diarylbenzidines and *N*,*N*-diaryldihydrophenazines were reported in [21]. By varying the reaction conditions and additional substituents in the phenyl rings, a possibility for the selective oxidation of *ortho*-(2-pyridyl)diphenylamine to the corresponding nitroxide, as well as the oxidation of both N-centers was demonstrated in [19]. The wide variety of the subsequent reaction channels for the radical cations formed under chemical or electrochemical oxidation of diarylamines, as well as availability of variously substituted diarylamines make them perspective starting compounds for organic synthesis.

In the present paper, convenient, easily reproducible, straightforward synthetic routes to *N*-arylpyridoindazolium salts were elaborated, based on both electrochemical and chemical (using bis(trifluoroacetoxy)iodobenzene, PIFA) oxidation of the *ortho*-pyridine-substituted diarylamines. Voltammetry studies of the electrochemical behavior of the novel pyridoindazolium salts and the starting diarylamines were performed confirming a possibility for their reversible redox interconversion.

## Results and Discussion

### Chemical oxidation

**Scheme 2:**
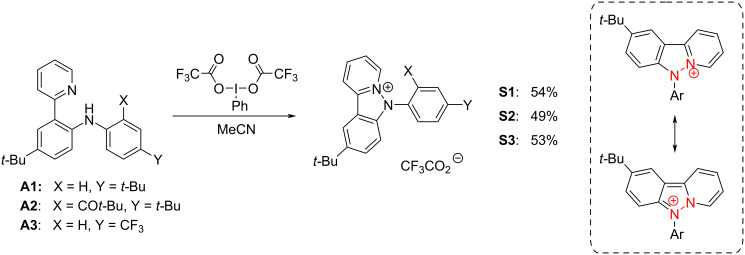
Synthesis of **S1**–**S3** salts using PIFA as an oxidant and the resonance structures demonstrating the electron density distribution in pyridoindazolium salts.

Three previously reported *ortho*-pyridine-substituted diarylamines containing electron-donating and electron-withdrawing groups were taken as the starting compounds (Scheme 2). As an oxidant, bis(trifluoroacetoxy)iodobenzene (PIFA) was used. It allowed obtaining the targeted heterocyclic cations in practical 49–54% yield for all starting diarylamines. Notably, a minor amount (5%) of the *N*,*N*’-diaryldihydrophenazine radical cation that is the byproduct corresponding to the intermolecular oxidative C–N coupling of the diarylamine **A1** was detected in the reaction mixture. This emphasizes that the both processes are of the same nature and proceed through the same intermediate (i.e., the diarylamines’ radical cation) and indicates the dominance of the intramolecular cyclization over the intermolecular C–N coupling process. Oxidation of diarylamines in the presence of an excess of trifluoroacetic acid gave no targeted pyridoindazolium salts, whereas the amount of diaryldihydrophenazine was increased (10%). Thus, protonation completely suppressed the intramolecular cyclization route.

Heterocyclic salts **S1**–**S3** were obtained as amorphous solids, soluble in polar solvents (acetonitrile, DMF, acetone) and chlorinated hydrocarbons (CHCl_3_, CH_2_Cl_2_).

The structure of new *N*-arylpyridoindazolium salts **S1**–**S3** was confirmed with HRMS and ^1^H, ^13^C and ^19^F NMR data; the complete assignment of the signals was performed using 2D NMR methods. The N–N bond formation was additionally confirmed via comparison of the ^1^H spectra for the salts and their diarylamine precursors. The absence of the signals corresponding to the acidic protons in the spectra of the salts excluded protonation of the pyridyl or amino groups. The downfield shift of the signals of both the *N*-aryl ring (for 0.5–1 ppm) and the pyridyl moiety (for more than 1 ppm) confirmed the positive charge delocalization over both N atoms and the involvement of the amines’ lone pair in the new 14-e aromatic system (Scheme 2).

One of the possible functionalities of the new compounds may be the utility of the charged aromatic fragment as an efficient leaving group in the S_N_(Ar) reactions, similarly to dibenzothiophenium triflates that have been recently reported as substrates for nucleophilic substitutions using potassium fluoride [22]. The study is in progress now.

### Voltammetry characterization of the *N*-arylpyridoindazolium salts **S1**–**S3** and their precursors, diarylamines **A1**–**A3**

**Figure 1:**
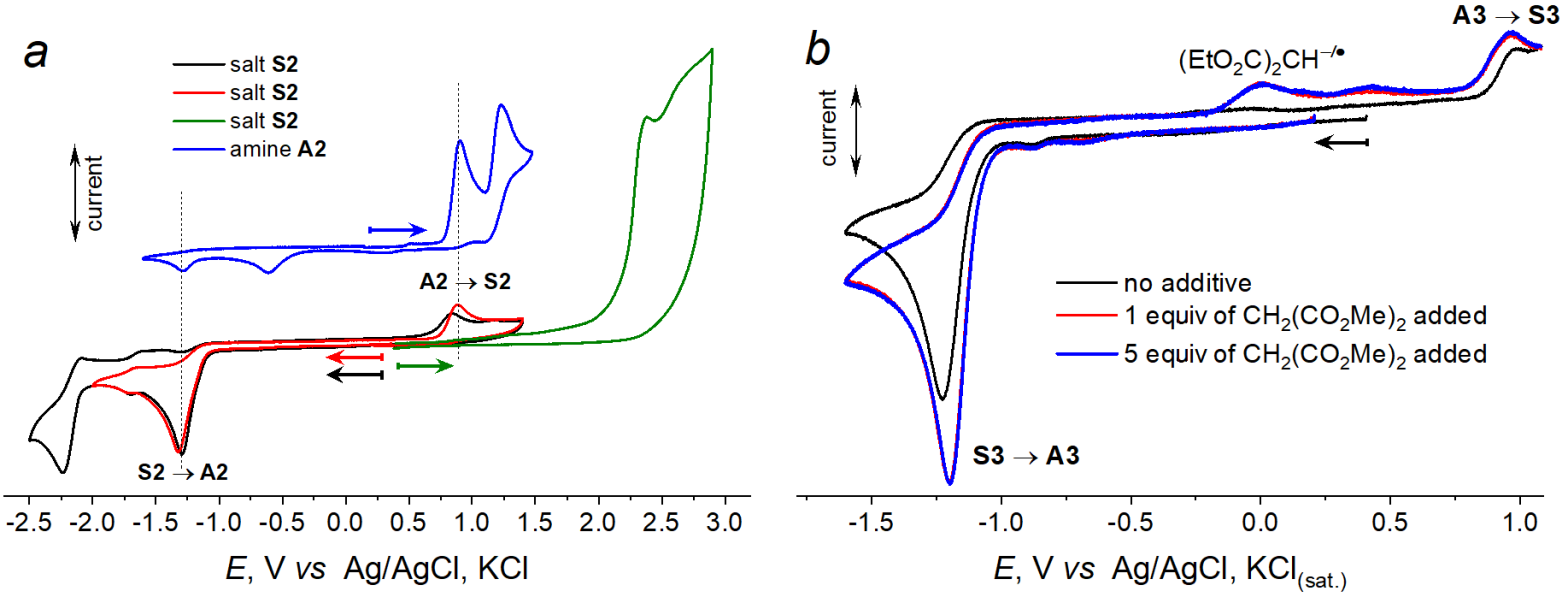
CV curves for salt **S2** and corresponding amine **A2** (left, Figure 1a) and salt **S3** with and without diethyl malonate additives (right, Figure 1b). (Pt, MeCN, 0.1 M Bu_4_NBF_4_, 0.1 V/s, vs Ag/AgCl, KCl_(sat.)_.

The electrochemical investigation of the new salts was performed at a Pt electrode in MeCN solution using Bu_4_NBF_4_ as a supporting electrolyte. Oxidation of the salts occurs at high positive potentials (>2 V vs Ag/AgCl, KCl_(sat.)_, Figure 1a), in accordance with the cationic nature of the heterocycle. In the negative potential range, an irreversible peak can be observed in the −1.2V to −1.3 V region (Table 1). In case of **S2**, the second reduction peak corresponds to the reduction of the carbonyl group. Thus, the electrochemical window for the new salts exceeds 3.5 V; that makes their molten forms perspective for application as ionic liquids.

**Table 1:** Peak potential values for the reduction of salts **S1**–**S3** (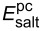, V) and oxidation of the corresponding diaryl amines **A1**–**A3** (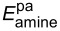).

Salt	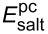 , V^a^	Amine	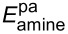 , V^b^

**S1**	−1.34	**A1**	1.03
**S2**	−1.29; −2.23	**A2**	1.07
**S3**	−1.23	**A3**	1.14

^a^In MeCN, 0.1 M Bu_4_NBF_4_, 0.1 V/s, glassy carbon disc electrode, vs Ag/AgCl, KCl_(sat.)_; ^b^in DMF, 0.1 M NaOTs, 0.1 V/s, Pt disc electrode, vs Ag/AgCl, KCl_(sat.)_

As follows from Table 1, oxidation of the starting amines **A1**–**A3** occurs at ca. 1–1.1 V vs Ag/AgCl, KCl_(sat.)_. The electrochemical study of PIFA reduction showed a broad irreversible peak with the onset potential value of +0.93 V (vs Ag/AgCl, KCl_(sat.)_). Thus, it is sufficiently strong to perform oxidation of diarylamines **A1**–**A3,** as it has been confirmed by the experimental results given in Scheme 2 above.

**Scheme 3:**
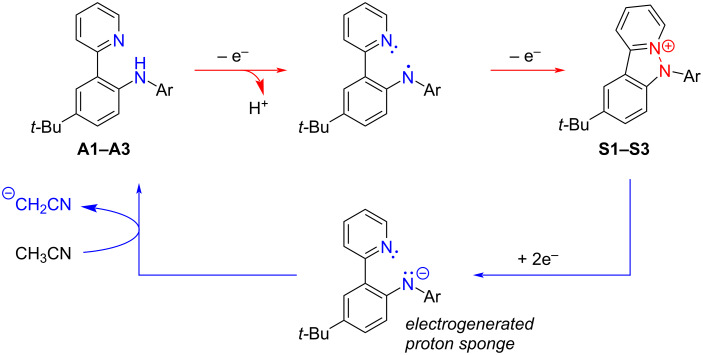
Redox-interconversion between diarylamines **A1**–**A3** and *N*-arylpyridoindazoliums **S1**–**S3**.

Comparison of the CV curves measured for *N*-arylpyridoindazolium salts **S1**–**S3** and their precursors – the diarylamines – sheds light of the nature of the electrochemical process. In Figure 1a, it is shown for salt **S2**. If the direction of the potential sweep is changed after passing the first reduction peak of salt **S2**, the new peak appears at the potential of 0.89 V that completely coincides with the first oxidation peak of the amine precursor. The CV curve for the diarylamine, in its turn, exhibited (in the reverse scan after oxidation of the amine) a new reduction peak that coincides to the first reduction peak of the pyridoindazolium salt (one more peak at less negative potential in the reverse scan after amine oxidation corresponds to the reduction of the protonated pyridyl moiety of the diarylamine). Thus, the voltammetry testing confirmed that oxidation of the pyridyl-containing amines results in the intramolecular heterocyclization yielding pyridoindazolium salts. The reduction of *N*-arylpyridoindazoliums in the presence of a source of protons restores the starting diarylamines. Redox-interconversion between diarylamines **A1**–**A3** and *N*-arylpyridoindazoliums **S1**–**S3** can be presented in the following scheme (Scheme 3).

Anions formed after two-electron reduction of *N*-arylpyridoindazolium salts are strong bases and can be considered as electrogenerated proton sponges. That was demonstrated taken salt **S3** as an example. Addition of relatively weak H-donors such as diethyl malonate significantly increases the reduction current of **S3** and shifts the potential value for 30 mV toward positive potentials (Figure 1b). In the reverse scan, a new peak appears, corresponding to oxidation of the malonate anion [23]; the peak of the amine oxidation is increased.

### Electrochemical synthesis of pyridoindazolium salts

**Scheme 4:**
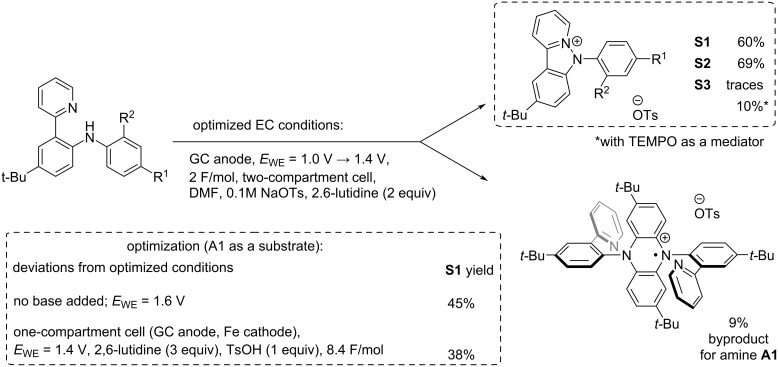
Electrochemical approach to pyridoindazolium salts.

The results of the voltammetry testing allowed to assume that pyridoindazolium salts can also be obtained using an anodic synthesis. Electric current as a reagent is inherently safe and easily scalable; electrosynthesis is in line with the green chemistry requirements [24]. To test the assumption, the preparative oxidation of amine **A1** was performed in the potentiostatic mode at 1.6 V (vs Ag/AgCl, KCl_(sat.)_) in a two-compartment cell in DMF; sodium tosylate was used as a supporting electrolyte. After a charge corresponding to 2 F per mole of amine **A1** was passed through the solution, the targeted salt **S1** was isolated in a promising 45% yield (Scheme 4).

**Figure 2:**
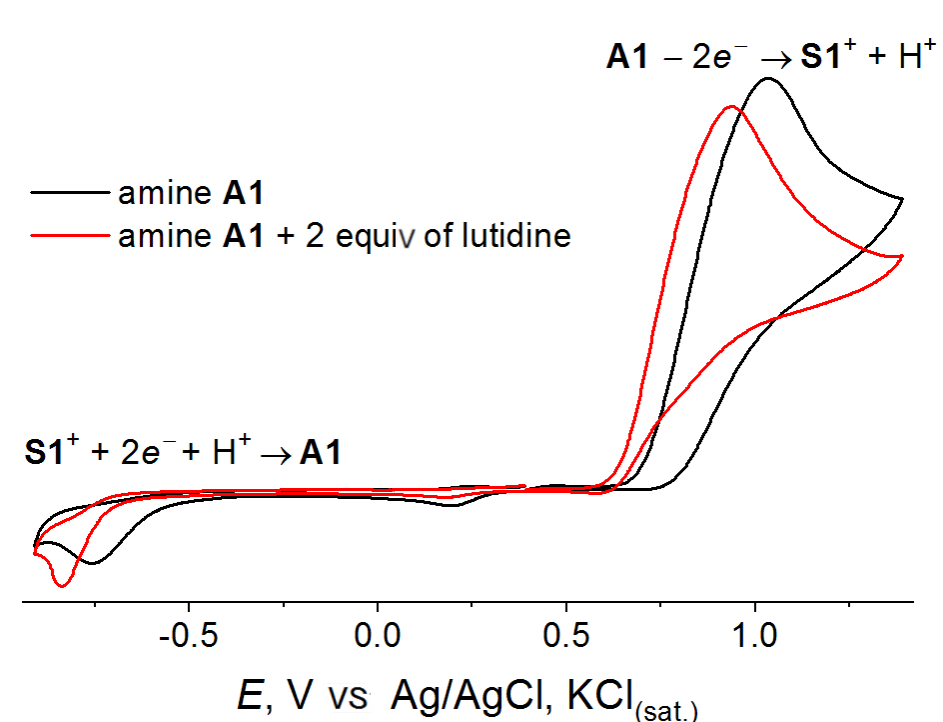
CV curves for amine **A1** without the lutidine additive (black curve) and after addition of 2 equiv (red curve). DMF, 0.1 M NaOTs, 0.1 V/s, Pt disc electrode, vs Ag/AgCl, KCl_(sat.)_

To find the optimal conditions, a base (2,6-lutidine) was added in the reaction mixture. A base is required to bind a proton released in the diarylamine oxidation. Our experiments with the acid additives (see above) showed that protonation of the pyridyl group suppresses the pyridoindazolium salt formation. Voltammetry testing also showed that 2,6-lutidine addition facilitates oxidation of the amine (Figure 2); the peak potential was of 100 mV shifted toward negative potentials. The pyridoindazolium salt reduction is also sensitive to the presence of a base since the electron transfer is followed by a protonation chemical step.

Indeed, a bulk electrolysis of amine **A1** in the presence of 2,6-lutidine gave much better (60%) yield of salt **S1**. Additionally, the electrolysis potential was decreased (since lutidine facilitates amines’ oxidation): the starting potential was set as 1.0 V and was gradually increased to 1.4 V. The attempt to perform the electrolysis in a one-compartment cell was unsuccessful: the yield of the salt dropped to 38%.

Thus, the optimized conditions of the potentiostatic electrolysis were the following: a two-compartment cell, a glassy carbon (GC) anode, DMF, the potential increased from 1.0 V to 1.4 V vs Ag/AgCl, KCl_(sat.)_), 2 F per mol of amine electricity passed, sodium tosylate (0.1 M) as a supporting electrolyte, and 2 equiv of 2.6-lutidine added. Bulk oxidation of amine **A2** under the optimized conditions gave the corresponding pyridoindazolium salt **S2** in a practical 69% yield.

It should be noted that a small amount of the oxidized *N*,*N*-diaryldihydrophenazine (5–10%) was also detected in the reaction mixture after the electrolysis of **A1**, similarly to the chemical oxidation. The radical cation of dihydrophenazine formed was isolated and studied using ESR and HRMS methods. The ESR spectrum (see Supporting Information File 1) was typical for this type of compounds: a characteristic quintet due to hyperfine splitting on two equivalent nitrogen atoms (a_N_ = 6.56 G) as well as additional triplet splitting provided by hyperfine interaction with a pair of equivalent protons (a_H_ = 1.89 G). In contrast, only traces of this admixture were detected for the diarylamines with electron-withdrawing substituents. This is in line with our previous mechanistic study [20–21] which revealed that the intermolecular C–N and C–C couplings are disfavored in case of the diarylamines with two electron-deficient phenyl rings.

To our surprise, the procedure did not work in the case of amine **A3**: only traces of pyridoindazolium salt **S3** were detected in the post-electrolysis mixture. Instead, a complex mixture of products was obtained. It looks as if the oxidation is non-selective; not a single reaction path dominates. This is typical for diarylamines containing electron-withdrawing substituents in both rings; commonly, electrochemical oxidation yields an inseparable mixture [21]. In case of amine **S2**, the electron-donating *tert*-butyl group in the *para*-position compensates the electron-withdrawing influence of the *ortho*-acyl substituent. This prevents formation of the phenazine byproduct (the *ortho*-position is occupied) in favor of the targeted process of the pyridoindazolium salt formation.

It is not entirely clear why the electrochemical approach does not work for amine **A3** in contrast to its chemical oxidation that was successful. Our experiments with the variation of the solvent (CH_3_CN and DMF), changing the supporting electrolyte (the replacement of NaOTs for more basic CF_3_CO_2_Na), addition of lutidine did not help.

**Figure 3:**
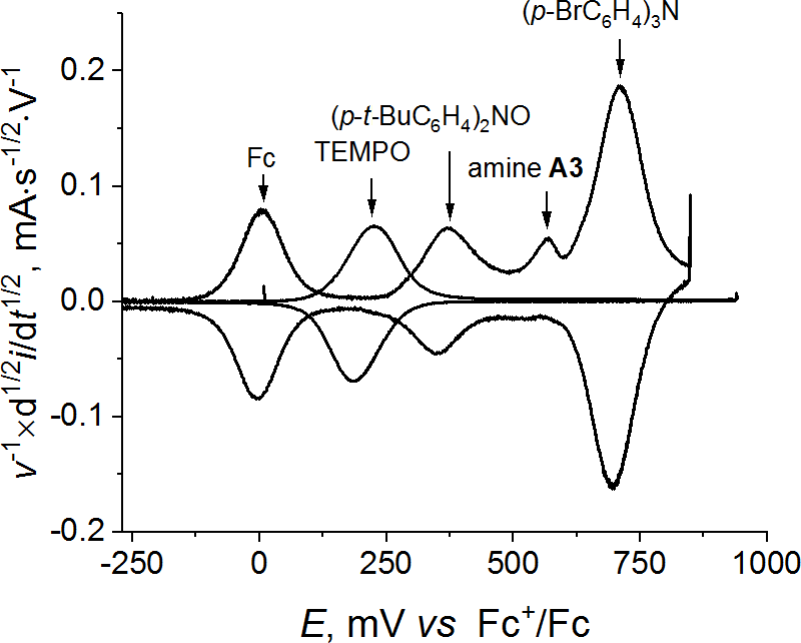
Semi-differential CV curves for the mediators (TEMPO, bis(4-*tert*-butylphenyl)nitroxide and tris(4-bromphenyl)amine) and amine **A3** in DMF/TsONa solution (0.1 V/s, Pt).

**Figure 4:**
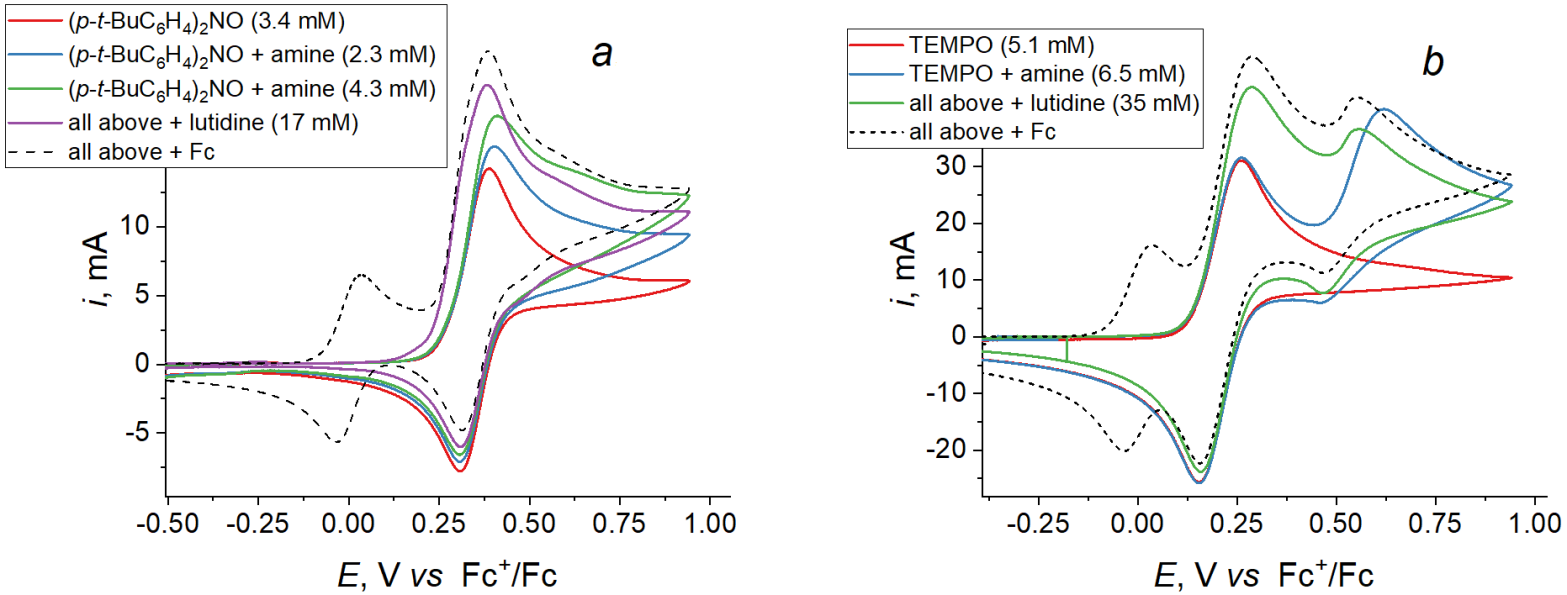
CV curves of bis(4-*tert*-butylphenyl)nitroxide (a) and TEMPO (b) with amine **A3** and 2,6-lutidine added (DMF/TsONa, 0.1 V/s, Pt).

To solve the problem, the mediatory oxidation of **A3** was also tried. Three possible mediators were tested: TEMPO, bis(4-*tert*-butylphenyl)nitroxide and tris(4-bromphenyl)amine. The voltammetry testing was performed in DMF using TsONa as a supporting electrolyte. As follows from Figure 3, the tertiary amine is inappropriate due to its too anodic oxidation potential whereas the two nitroxide radicals might be suitable. Indeed, an increase in the oxidation current of a mediator was observed in both cases after **A3** has been added into the reaction mixture (Figure 4a,b). Notably, the effect was more pronounced in the presence of lutidine, especially in the case of TEMPO. The difference between the oxidation potential of **A3** and the potential of the TEMPO/TEMPO^+^ redox couple is rather significant (ca. 0.35 V); the base additives make oxidation of amines less anodic (due to the H-bonding and facilitation of deprotonation of the radical cations), thus narrowing the potential gap.

Preparative experiments were performed under the same conditions as described above: A one-compartment cell, DMF, 0.25 mol equiv of a mediator was added. Potentiostatic electrolyses were carried out at the potential of the mediator oxidation. The current was dropped down after ca. 1.5 equivalents of electricity has been passed through the solution. Analysis of the reaction mixtures showed that in both cases a certain amount of the target salt **S3** was formed. When TEMPO was used as a mediator, **S3** was isolated in 10% yield, along with the starting amine (18%) and the tetraarylhydrazine as the main product (50%). In the case of the diarylnitroxide, a complicated multicomponent reaction mixture was formed. Besides the products mentioned above, it contained some other compounds, which have not been identified.

Among the amines studied, amine **A3** is less prone to oxidation (due to the electron-withdrawing effect of the trifluoromethyl group) and is the less basic; the presence of a lutidine base even more fastens deprotonation of the radical cation formed in oxidation. Since oxidation occurs in the bulk and the potential of the mediator is insufficient for the further oxidation of the electrophilic CF_3_-substituted diarylaminyl radicals to the corresponding cations, the N–N coupling of thus formed aminyl radicals dominates over the intramolecular cyclization. This oxidation path is inherent to non-bulky amines of low basicity in the presence of a base [20,25]. In our case, the base is required to prevent acidification of the reaction mixture.

## Conclusion

A novel facile approach to *N*-arylpyridoindazolium salts was proposed, based on direct oxidation of the *ortho*-pyridine-substituted diarylamines. The oxidation can be performed either chemically, using bis(trifluoroacetoxy)iodobenzene as the oxidant, or electrochemically, via potentiostatic oxidation. The electrochemical synthesis occurs under mild conditions; no chemical reagents are required except electric current. Both approaches can be considered as a late-stage functionalization; the easily available *ortho*-pyridyl-substituted diarylamines are used as the precursors. The direct approaches to *N*-arylpyridoindazolium salts elaborated herein open a route to broadening a scope of these practically useful compounds with multiple functionalities that were poorly available previously.

## Supporting Information

File 1 Analytical data, NMR and MS spectra.

## Data Availability

All data that supports the findings of this study is available in the published article and/or the supporting information to this article.
