# Supplementary material for: Novel oxidative routes to N-arylpyridoindazolium salts
Source: Beilstein J Org Chem. 2024 Aug 7;20:1906–13. doi: 10.3762/bjoc.20.166 (PMC11318609; doi:10.3762/bjoc.20.166)
Supplement: File 1 — Analytical data, NMR and MS spectra. [file Beilstein_J_Org_Chem-20-1906-s001.pdf]

BEILSTEIN JOURNAL OF ORGANIC CHEMISTRY

# Supporting Information

for

# Novel oxidative routes to *N*-arylpyridoindazolium salts

Oleg A. Levitskiy, Yuri K. Grishin and Tatiana V. Magdesieva

*Beilstein J. Org. Chem.* **2024,** *20,* 1906–1913. doi:10.3762/bjoc.20.166

# Analytical data, NMR and MS spectra

License and Terms: This is a supporting information file under the terms of the Creative Commons Attribution License (https://creativecommons.org/licenses/by/4.0). Please note that the reuse, redistribution and reproduction in particular requires that the author(s) and source are credited and that individual graphics may be subject to special legal provisions.
The license is subject to the *Beilstein Journal of Organic Chemistry* terms and conditions: (https://www.beilstein-journals.org/bjoc/terms)

## Contents

*1. Experimental details* ... S3

*2. Electrosynthesis of pyridoindazolium trifluoroacetates* **$S1^+CF_3CO_2^–$** *and* **$S2^+CF_3CO_2^–$** *(general procedure)* ... S4

*3. Synthesis of pyridoindazolium trifluoroacetates* **$S1^+CF_3CO_2^–$**, **$S2^+CF_3CO_2^–$** *and* **$S3^+CF_3CO_2^–$** *via oxidation of the amines by PIFA (general procedure)* ... S4

*4. Experimental protocol for mediated anodic oxidation of amine* **A3** ... S4

*5. Characterization of the new compounds* ... S6

4.1 Analytical data for **$S1^+OTs^–$** and **$S1^+CF_3CO_2^–$** ... S6

4.2 Analytical data for **$S2^+OTs^–$** and **$S2^+CF_3CO_2^–$** ... S6

4.3 Analytical data for **$S3^+CF_3CO_2^–$** and **$S3^+OTs^–$** ... S7

4.4 Characterization of diaryldihydrophenazinium **$1Phz^+OTs^–$** radical cation ... S7

*6. Copies of the spectra* ... S8

6.1 **$^1$H NMR** spectrum of **$S1^+OTs^–$** ... S8

6.2 **$^{13}$C NMR** spectrum of **$S1^+OTs^–$** ... S8

6.3 **HSQC** spectrum of **$S1^+OTs^–$** ... S9

6.4 HMBC spectrum of **$S1^+OTs^–$** ... S10

6.5 **$^1$H NMR** spectrum of **$S1^+CF_3CO_2^–$** ... S11

6.6 **$^1$H NMR** spectrum of **$S2^+CF_3CO_2^–$** ... S12

6.7 **$^{13}$C NMR** spectrum of **$S2^+CF_3CO_2^–$** ... S12

6.8 **$^{19}$F NMR** spectrum of **$S2^+CF_3CO_2^–$** ... S13

6.9 **COSY** spectrum of **$S2^+CF_3CO_2^–$** ... S13

6.10 **HSQC** spectrum of **$S2^+CF_3CO_2^–$** ... S14

6.11 **HMBC** spectrum of **$S2^+CF_3CO_2^–$** ... S15

6.12 **$^1$H NMR** spectrum of **$S2^+OTs^–$** ... S17

6.13 **$^{13}$C NMR** spectrum of **$S2^+OTs^–$** ... S17

6.14 **$^1$H NMR** spectrum of **$S3^+CF_3CO_2^–$** ... S18

6.15 **$^{13}$C NMR** spectrum of **$S3^+CF_3CO_2^–$** ... S18

6.16 **$^{19}$F NMR** spectrum of **$S3^+CF_3CO_2^–$** ... S19

6.17 **HSQC** spectrum of **$S3^+CF_3CO_2^–$** ... S19

6.18 **HMBC** spectrum of **$S3^+CF_3CO_2^–$** ... S20

6.19 **$^1$H NMR** spectrum of a mixture of amine **A3** and its dimer (tetraarylhydrazine) obtained from the TEMPO-mediated electrolysis ... S21

6.20 **$^1$H NMR** spectrum of **$S3^+OTs^–$** obtained from the TEMPO-mediated electrosynthesis ... S21

6.21 **GC-MS** analysis results for a mixture of amine **A3** and its dimer (tetraarylhydrazine) obtained from the TEMPO-mediated electrolysis ... S22

Chromatogram: ... S22

6.22 **ESI-HRMS** spectrum of **S1$^+$CF$_3$CO$_2$$^-$** ........ S22
6.23 **ESI-HRMS** spectrum of **S2$^+$CF$_3$CO$_2$$^-$** ........ S23
6.24 **ESI-HRMS** spectrum of **S3$^+$CF$_3$CO$_2$$^-$** ........ S23
6.25 **ESI-HRMS** spectrum of diaryldihidrophenazinium tosylate **1Phz$^+$OTs$^-$** ........ S24
7. CV of bis(trifluoroacetoxy)iodobenzene (PIFA) ........ S25
8. Cyclic voltammpgram of **S1$^+$OTs$^-$** ........ S25

## 1. *Experimental details*

$^1H$ (400.0 MHz) and $^{13}C$ (100.6 MHz) NMR spectra (including COSY, HMBC, HSQC) were recorded using an Aglient 400-MR spectrometer in $CDCl_3$. Chemical shifts were referenced to the nondeuterated aliquot of the solvent.

*Mass spectra*. $CH_3CN$ (LC–MS grade) for ESI–MS experiments was ordered from Merck and used as received. Sodium formate (for HPLC) for calibration was ordered from Sigma-Aldrich. The samples for ESI–MS experiments were prepared in 1.8 mL glass vials/screw top caps with PTFE septa for HPLC experiments (Agilent Technologies).

*Preparative electrolysis* was performed with AutoLab PGSTAT100N potentiostat in a two-compartment or one-compartment cell of 10 mL volume. The WE was glassy carbon plate (300 $mm^2$); the CE was a stainless steel wire. The solution was stirred magnetic stirring bar and deaerated with an argon flow.

*Voltammetric experiments* were performed with Biologic BP-300 potentiostat, in a ALS Co. three-electrode cell of 2 mL with a platinum wire counter electrode (CE) and anhydrous Ag/0.01 M $AgNO_3$ (MeCN) reference electrode (RE). Ferrocene was used as internal standard in each experiment and all measured potentials were converted to the Ag/AgCl,KCl(sat.) reference electrode (in the latter scale, the potential for the $Fc^{0/+}$ redox couple is equal to 0.475 V in acetonitrile). A Pt disk electrode with active surface area of 0.077 $cm^2$ was used as the working electrode (WE). Hardware ohmic drop compensation was employed. All solutions were thoroughly deaerated by passing an argon flow through the solution prior to the CV experiments and above the solution during the measurements, the supporting electrolyte in all experiments was 0.1 M *n*-$Bu_4NBF_4$ (Aldrich, purity > 99%), which has been recrystallized from water and dried by heating at 150 °C under reduced pressure (0.05 Torr) prior to use. Acetonitrile (82 °C / 1 atm) and *N*,*N*-dimethylformamide (46 °C / 16 mbar) were distilled over $P_2O_5$ and stored under argon.

*The ESR spectra* were recorded with the X-band Bruker EMX-plus spectrometer for solutions deaerated using a standard freeze-pump-thaw technique (concentrations of radicals were 0.2– 0.4 mM). The ESR spectra of the radicals' solutions were registered at 290 K with 1 mW microwave power and 0.5 G modulation amplitude.

Solvents used for synthesis and chromatography were purified prior to experiments according to standard procedures.[1]

(Bis(trifluoroacetoxy)iodo)benzene (PIFA) was synthesized according to literature procedure [2].

Sodium tosylate was prepared by neutralization of *p*-toluenesulfonic acid aqueous solution by sodium hydroxide, evaporation of the resulting solution and purification by crystallization from ethanol. Crystallization was performed as follows. Crude solid NaOTs were extracted by ethanol in Soxhlet apparatus. The resulting mixture of saturated NaOTs solution and crystalline NaOTs was cooled to −20 °C, filtered and dried under reduced pressure (0.05 Torr).

Previously described amines **A1** [3], **A2** [4] and **A3** [5] were prepared according to literature procedure [4] with yields 87%, 96% and 95%, respectively. All other reactants were commercially available and used as recived.


1. Armarego, W. L. F. *Purification of Laboratory Chemicals*, 8th ed.; Butterworth-Heinemann, 2017
2. Kazmierczak, P.; Skulski, L. *Molecules* **2002**, *7*, 810–812. doi:10.3390/71100810
3. Levitskiy, O. A.; Klimchuk, I. A.; Grishin, Y. K.; Roznyatovsky, V. A.; Tarasevich, B. N.; Magdesieva, T. V. *Synthesis* **2022**, *54*, 1601–1612. doi:10.1055/a-1683-0315
4. Levitskiy, O. A.; Grishin, Y. K.; Magdesieva, T. V. *Mendeleev Commun.* **2024**, *34*, 251–254. doi:10.1016/j.mencom.2024.02.029
5. Dulov, D. A.; Magdesieva, T. V. *J. Org. Chem.* **2023**, *88*, 12765–12775. doi:10.1021/acs.joc.3c01445

2. *Electrosynthesis of pyridoindazolium trifluoroacetates* **$S1^+CF_3CO_2^-$** *and* **$S2^+CF_3CO_2^-$** *(general procedure)*

A two-compartment cell (electrode compartments separated by a glass frit) was filled with 13 ml of 0.1 M solution of sodium tosylate in dry DMF (4 ml in the cathodic compartment and 9 ml in the anodic one). *ortho*-(2-Pyridyl)diarylamine (0.25 mmol) and 2,6-lutidine (60 μl, 0.5 mmol) were added to the working electrode (glassy carbon anode) compartment and 0.1 ml of nitrobenzene was added to the counter electrode (stainless steel cathode) compartment as a depolarizer. The potential was gradually increased from 0.6 to 1 V vs. $Ag^+/Ag$ (1.0 V to 1.4 V vs. Ag/AgCl, $KCl_{(sat.)}$) and 2 F of electricity per mole of the substrate was passed. The anolyte was separated and evaporated. The residue was dissolved in $CH_2Cl_2$, washed with water, evaporated and dried in vacuum to dryness. The targeted pyridoindazolium salt was isolated by column chromatography on silica gel. Gradient elution from $CHCl_3$/acetone 5:1 to pure acetone was performed first to remove byproducts (fraction containing diaryldihydrophenazinium radical cation was eluted by acetone). Further elution with $CHCl_3$/MeOH 1:1 yields fractions containing the targeted pyridoindazolium salt. Fractions containing pyridoindazolium salt were evaporated, redissolved in $CH_2Cl_2$, filtered through celite (to remove silica gel particles) and evaporated to dryness.

Oxidation of 90 mg (0.25 mmol) of amine **A1** results in 80 mg (60%) of **$S1^+OTs^-$** as a gray powder and 10 mg (9%) of **$1Phz^+OTs^-$** as a dark-green powder.

Oxidation of 75 mg (0.17 mmol) of amine **A2** results in 72 mg (69%) of **$S2^+OTs^-$** as a light beige powder.

Analytical data for the new compounds see in section 4.

3. *Synthesis of pyridoindazolium trifluoroacetates* **$S1^+CF_3CO_2^-$**, **$S2^+CF_3CO_2^-$** *and* **$S3^+CF_3CO_2^-$** *via oxidation of the amines by PIFA (general procedure)*

A solution of 1 mmol of the *ortho*-(2-pyridyl)diarylamine in 2.2 ml of acetonitrile in a 20 ml vial was placed into a water bath and 1 mmol of (bis(trifluoroacetoxy)iodo)benzene (PIFA) powder was added with stirring. The mixture was stirred for 1.5 h and then diluted with saturated $NaHCO_3$ aqueous solution and extracted with $CH_2Cl_2$. Organic layer was evaporated under reduced pressure and the residue was dried under vacuum (0.1 mmHg). The targeted pyridoindazolium salt was isolated by column chromatography (silica gel; gradient elution from $CHCl_3$/acetone 1:1 to pure acetone to remove byproducts and further elution with $CHCl_3$/MeOH 1:1 to collect fractions of the targeted pyridoindazolium salt). Fractions containing pyridoindazolium salt were evaporated, redissolved in $CH_2Cl_2$, filtered through celite (to remove silica gel particles) and evaporated to dryness.

**$S1^+CF_3CO_2^-$**: Yield 97 mg (54%) starting from 137 mg (0.38 mmol) of amine **A1**.

**$S2^+CF_3CO_2^-$**: Yield 198 mg (49%) starting from 323 mg (0.73 mmol) of amine **A2**.

**$S3^+CF_3CO_2^-$**: Yield 115 mg (53%) starting from 165 mg (0.45 mmol) of amine **A3**.

4. *Experimental protocol for mediated anodic oxidation of amine* **A3**

A two-compartment cell (electrode compartments separated by a glass frit) was filled with 11 ml of 0.1 M solution of sodium tosylate in dry DMF (3 ml in the cathodic compartment and 8 ml in the anodic one). Amine **A3** (86 mg, 0.24 mmol) and 2,6-lutidine (58 μl, 0.48 mmol) were added to the working electrode (glassy carbon anode) compartment and 0.1 ml of nitrobenzene was added to the counter electrode (stainless steel cathode) compartment as a depolarizer. The potential was gradually increased from 0.3 to 0.5 V vs $Ag^+/Ag$ (0.7 V to 0.9 V vs Ag/AgCl, $KCl_{(sat.)}$) and 1.5 F of electricity per mole of the substrate was passed. The anolyte was separated and evaporated. The

residue was dissolved in $CH_2Cl_2$, washed with water, evaporated and dried in vacuum to dryness. The residue was subjected to column chromatography on silica gel. Gradient elution from $CHCl_3$/acetone 5:1 to pure acetone was performed first and afforded the fraction composed of the starting amine **A3** (0.04 mmol, 18% of the initial quantity) and its dimer – the tetraarylhydrazine (0.06 mmol, 50% yield). Further elution with $CHCl_3$/MeOH 1:1 yields fraction containing the targeted pyridoindazolium salt. Fractions containing pyridoindazolium salt were evaporated, redissolved in $CH_2Cl_2$, filtered through celite (to remove silica gel particles) and evaporated to dryness, yielding **S3$^+$OTs$^-$** (15 mg, 10% yield)).

*5. Characterization of the new compounds*

4.1 Analytical data for **S1$^+$OTs$^-$** and **S1$^+$CF$_3$CO$_2$$^-$**

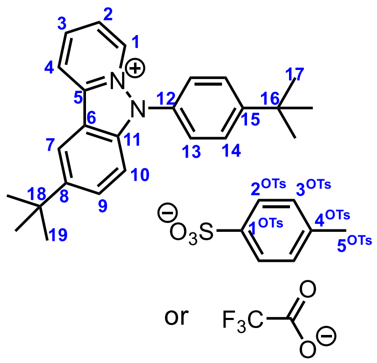


**$^{1}$H NMR** of **S1$^+$OTs$^-$** (400 MHz, CDCl$_3$) δ 8.86 (ddd, $^3$J = 8.6, $^4$J = 1.4, $^5$J = 0.9 Hz, 1H, **H-4**), 8.83 (ddd, $^3$J = 6.9, $^4$J = 1.0, $^5$J = 0.9 Hz, 1H, **H-1**), 8.40 (dd, $^4$J = 1.8, $^5$J = 0.7 Hz, 1H, **H-7**), 8.31 (ddd, $^3$J = 8.6, $^3$J = 7.4, $^4$J = 1.0 Hz, 1H, **H-3**), 8.09 (ddd, $^3$J = 7.4, $^3$J = 6.9, $^4$J = 1.4 Hz, 1H, **H-2**), 7.88 (dd, $^3$J = 9.0, $^4$J = 1.8 Hz, 1H, **H-9**), 7.75 – 7.71 (m$_{AA'BB'}$, 2H, **H-14**), 7.67 – 7.62 (m$_{AA'BB'}$, 2H, **H-2$^{OTs}$**), 7.62 – 7.57 (m$_{AA'BB'}$, 2H, **H-13**), 7.29 (dd, $^3$J = 9.0, $^5$J = 0.7 Hz, 1H, **H-10**), 7.00 – 6.96 (m$_{AA'BB'}$, 2H, **H-3$^{OTs}$**), 2.23 (s, 3H, **H-5$^{OTs}$**), 1.42 (s, 9H, **H-19**), 1.39 (s, 9H, **H-17**).

**$^{13}$C NMR** of **S1$^+$OTs$^-$** (101 MHz, CDCl$_3$) δ 156.43 (**C15**), 148.94 (**C8**), 144.36 (**C1$^{OTs}$**), 138.70 (**C11**), 138.37 (**C4$^{OTs}$**), 137.12 (**C5**), 135.14 (**C3**), 132.73 (**C9**), 128.73 (**C14**), 128.46 (**C12**), 128.35 (**C13**), 128.18 (**C3$^{OTs}$**), 126.07 (**C2$^{OTs}$**), 125.43 (**C1**), 123.31 (**C2**), 120.57 (**C4**), 118.01 (**C7**), 115.36 (**C6**), 109.70 (**C10**), 35.33 (**C18**), 35.31 (**C16**), 31.45 (**C19**), 31.15 (**C17**), 21.22 (**C5$^{OTs}$**).

**$^{1}$H NMR** of **S1$^+$CF$_3$CO$_2$$^-$** (400 MHz, CDCl$_3$) δ 8.87 (dt, $^3$J = 7.0, $^4$J = 1.0 Hz, $^5$J = 0.9 Hz, 1H, **H-1**), 8.81 (ddd, $^3$J = 8.6, $^4$J = 1.4, $^5$J = 0.9 Hz, 1H, **H-4**), 8.39 (dd, $^4$J = 1.8, $^5$J = 0.8 Hz, 1H, **H-7**), 8.31 (ddd, $^3$J = 8.6, $^3$J = 7.4, $^4$J = 1.0 Hz, 1H, **H-3**), 8.08 (ddd, $^3$J = 7.4, $^3$J = 7.0, $^4$J = 1.4 Hz, 1H, **H-2**), 7.93 (dd, $^3$J = 9.0, $^4$J = 1.8 Hz, 1H, **H-9**), 7.82 – 7.78 (m, 2H, **H-14**), 7.63 – 7.59 (m, 2H, **H-13**), 7.34 (dd, $^3$J = 9.0, $^5$J = 0.8 Hz, 1H, **H-10**), 1.46 (s, 9H, **H-19**), 1.43 (s, 9H, **H-17**).

**HRMS (ESI):** m/z 357.2329 ([M]$^+$,357.2325 calc. for $C_{25}H_{29}N_2^+$)

4.2 Analytical data for **S2$^+$OTs$^-$** and **S2$^+$CF$_3$CO$_2$$^-$**

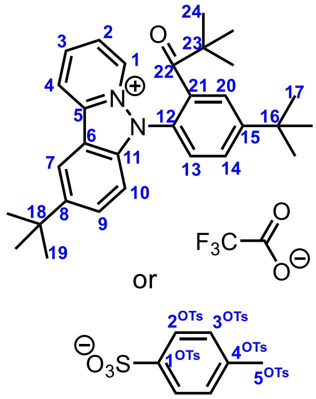


**$^{1}$H NMR** spectrum of **S2$^+$CF$_3$CO$_2$$^-$**: (400 MHz, CDCl$_3$): δ 8.68 (d, $^3$J = 8.6 Hz, 1H, **H-4**), 8.60 (d, $^3$J = 7.0 Hz, 1H, **H-1**), 8.34 – 8.27 (m, 2H, **H-3,7**), 7.91 (ddd, $^3$J = 7.3, $^3$J = 7.0, $^4$J = 1.3 Hz, 1H, **H-2**), 7.87 (dd, $^3$J = 8.9, $^4$J = 1.8 Hz, 1H, **H-9**), 7.85 (dd, $^3$J = 8.4, $^4$J = 2.2 Hz, 1H, **H-14**), 7.78 (d, $^4$J = 2.2 Hz, 1H, **H-20**), 7.64 (d, $^3$J = 8.4 Hz, 1H, **H-13**), 7.19 (dd, $^3$J = 8.9, $^5$J = 0.5 Hz, 1H, **H-10**), 1.43 (s, 9H, **H-19**), 1.42 (s, 9H, **H-17**), 1.03 (s, 9H, **H-24**).

**$^{13}$C NMR** spectrum of **S2$^+$CF$_3$CO$_2$$^-$** (101 MHz, CDCl$_3$): δ 209.56 (**C22**), 161.78 (q, $^2$J$_{CF}$ = 35 Hz, CF$_3$*C*O$_2$$^-$), 156.64 (**C15**), 149.08 (**C8**), 139.66 (**C21**), 139.45 (**C11**), 137.54 (**C5**), 135.77 (**C3**), 132.58 (**C9**), 131.85 (**C13**), 130.53 (**C14**), 126.79 (**C1**), 126.23 (**C12**), 125.70 (**C20**), 122.84 (**C2**), 119.89 (**C4**), 117.99 (**C7**), 116.64 (q, $^1$J$_{CF}$ = 293 Hz, *C*F$_3$CO$_2$$^-$), 115.94 (**C6**), 109.84 (**C10**), 44.91 (**C23**), 35.60 (**C16**), 35.36 (**C18**), 31.45 (**C19**), 31.08 (**C17**), 27.76 (**C24**).

**$^{19}$F NMR** spectrum of **S2$^+$CF$_3$CO$_2$$^-$** (376 MHz, CDCl$_3$) δ −75.62.

**$^{1}$H NMR** spectrum of **S2$^+$OTs$^-$** (400 MHz, CDCl$_3$): δ 8.83 (d, $^3$J = 8.6 Hz, 1H, **H-4**), 8.65 (d, $^3$J = 7.0 Hz, 1H, **H-1**), 8.39 (d, $^4$J = 1.0 Hz, 1H, **H-7**), 8.31 (ddd, $^3$J =8.6, $^3$J =7.3, $^4$J =0.8, 1H, **H-3**), 7.95 (ddd, $^3$J = 7.3, $^3$J = 7.0, $^4$J = 1.2 Hz, 1H, **H-2**), 7.86 (dd, J = 8.9, 1.7 Hz, 1H, **H-9**), 7.82 – 7.80 (m, 2H, **H-13,14**), 7.77 – 7.75 (m, 1H, **H-20**), 7.68 – 7.63 (m, 2H, **H-2$^{OTs}$**), 7.16 (d, $^3$J = 8.9 Hz, 1H, **H-10**), 7.03 – 6.97 (m, 2H, **H-3$^{OTs}$**), 2.25 (s, 3H, **H-5$^{OTs}$**), 1.42 (s, 9H, **H-19**), 1.42 (s, 9H, **H-17**), 1.01 (s, 9H, **H-24**).

**$^{13}$C NMR** spectrum of **S2$^+$OTs$^-$** (101 MHz, CDCl$_3$): δ 209.50 (**C22**), 156.45 (**C15**), 149.04 (**C8**), 144.71 (**C1$^{OTs}$**), 139.61 (**C21**), 139.39 (**C11**), 138.29 (**C4$^{OTs}$**), 137.51 (**C5**), 135.56 (**C3**), 132.55 (**C9**), 132.48 (**C13**), 130.47 (**C14**), 128.23 (**C3$^{OTs}$**), 127.15 (**C1**), 126.30 (**C12**), 126.21 (**C2$^{OTs}$**),

125.57 (**C20**), 122.68 (**C2**), 120.34 (**C4**), 118.38 (**C7**), 116.05 (**C6**), 109.72 (**C10**), 44.83 (**C23**), 35.59 (**C16**), 35.39 (**C18**), 31.53 (**C19**), 31.15 (**C17**), 27.82 (**C24**), 21.34 (**C5$^{OTs}$**).

**HRMS (ESI):** m/z 441.2897 ($[M]^+$, 441.2900 calc. for $C_{30}H_{37}N_2O^+$)

### 4.3 Analytical data for $\mathbf{S3^+CF_3CO_2^-}$ and $\mathbf{S3^+OTs^-}$

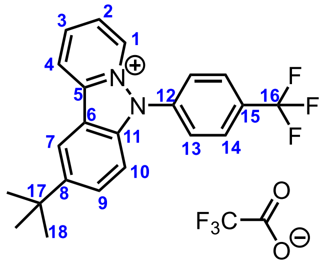

**$^1$H NMR** spectrum of $\mathbf{S3^+CF_3CO_2^-}$ (400 MHz, $CDCl_3$): δ 9.12 (d, $^3J$ = 7.0 Hz, 1H, **H-1**), 8.77 (d, $^3J$ = 8.6 Hz, 1H, **H-4**), 8.37 (d, $^4J$ = 1.8 Hz, 1H, **H-7**), 8.28 (dd, $^3J$ = 8.6, 7.3 Hz, 1H, **H-3**), 8.09 – 8.03 (m, 4H, **H-13**,**14**), 8.00 (ddd, $^3J$ = 7.3, 7.0 Hz, $^4J$ = 1.2 Hz, 1H, **H-2**), 7.95 (dd, $^3J$ = 9.0, $^4J$ = 1.8 Hz, 1H, **H-9**), 7.32 (d, $^3J$ = 9.0 Hz, 1H, **H-10**), 1.45 (s, 9H, **H-18**).

**$^{13}$C NMR** spectrum of $\mathbf{S3^+CF_3CO_2^-}$ (101 MHz, $CDCl_3$): δ 160.87 (q, $^2J_{CF}$ = 33 Hz, $CF_3\mathit{C}O_2^-$), 149.60 (**C8**), 138.58 (**C11**), 137.75 (**C5**), 135.57 (**C3**), 134.69, 134.47 (q, $^2J_{CF}$ = 34 Hz, **C15**) 133.29 (**C9**), 129.83 (**C13**), 128.98 (q, $^3J_{CF}$ = 3.3 Hz, **C14**), 126.52 (**C1**), 123.35 (**C2**), 123.19 (q, $^1J_{CF}$ = 273 Hz, **C16**), 120.19 (**C4**), 117.95 (**C7**), 117.06 (q, $^1J_{CF}$ = 296 Hz, $\mathit{C}F_3CO_2^-$ Hz), 115.70 (**C6**), 109.65 (**C10**), 35.44 (**C17**), 31.46 (**C18**).

**$^{19}$F NMR** spectrum of $\mathbf{S3^+CF_3CO_2^-}$ (376 MHz, $CDCl_3$): δ −63.06 (Ar-$CF_3$), −75.16 ($CF_3CO_2^-$).

**$^1$H NMR** spectrum of $\mathbf{S3^+OTs^-}$ (400 MHz, $CDCl_3$): δ 9.16 (d, $^3J$ = 6.9 Hz, 1H, **H-1**), 8.67 (d, $^3J$ = 8.4 Hz, 1H, **H-4**), 8.33 (d, $^4J$ = 1.9 Hz, 1H, **H-7**), 8.26 – 8.20 (m, 1H, **H-3**), 8.04 – 8.00 (m, 2H, **H-13** or **14**), 8.00 – 7.96 (m, 2H, **H-13** or **14**), 7.93 (dd, $^3J$ = 9.0, $^4J$ = 1.9 Hz, 1H, **H-9**), 7.93 – 7.86 (m, 1H, **H-2**), 7.65 – 7.62 (m, 2H, **H-2$^{OTs}$**), 7.30 (d, $^3J$ = 9.0 Hz, 1H, **H-10**), 7.08 – 7.03 (m, 2H, **H-3$^{OTs}$**), 2.30 (s, 3H, **Me$^{OTs}$**), 1.45 (s, 9H, **H-18**).

**HRMS (ESI):** m/z 369.1579 ($[M]^+$, 369.1573 calc. for $C_{22}H_{20}N_2F_3^+$)

### 4.4 Characterization of diaryldihydrophenazinium $\mathbf{1Phz^+OTs^-}$ radical cation

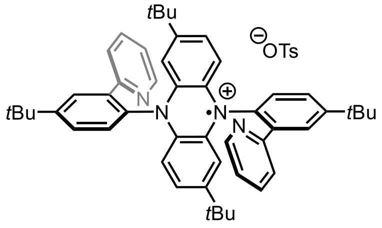

**HRMS (ESI):** m/z 712.4500 ($[M]^+$, 712.4499 calc. for $C_{50}H_{56}N_4^+$)

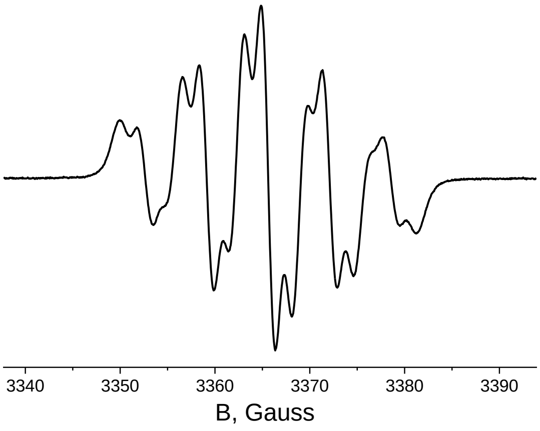


ESR spectrum of $\mathbf{1Phz^+OTs^-}$ in toluene

## 6. *Copies of the spectra*

### 6.1 $^{1}$**H NMR** spectrum of **S1$^{+}$OTs$^{-}$**

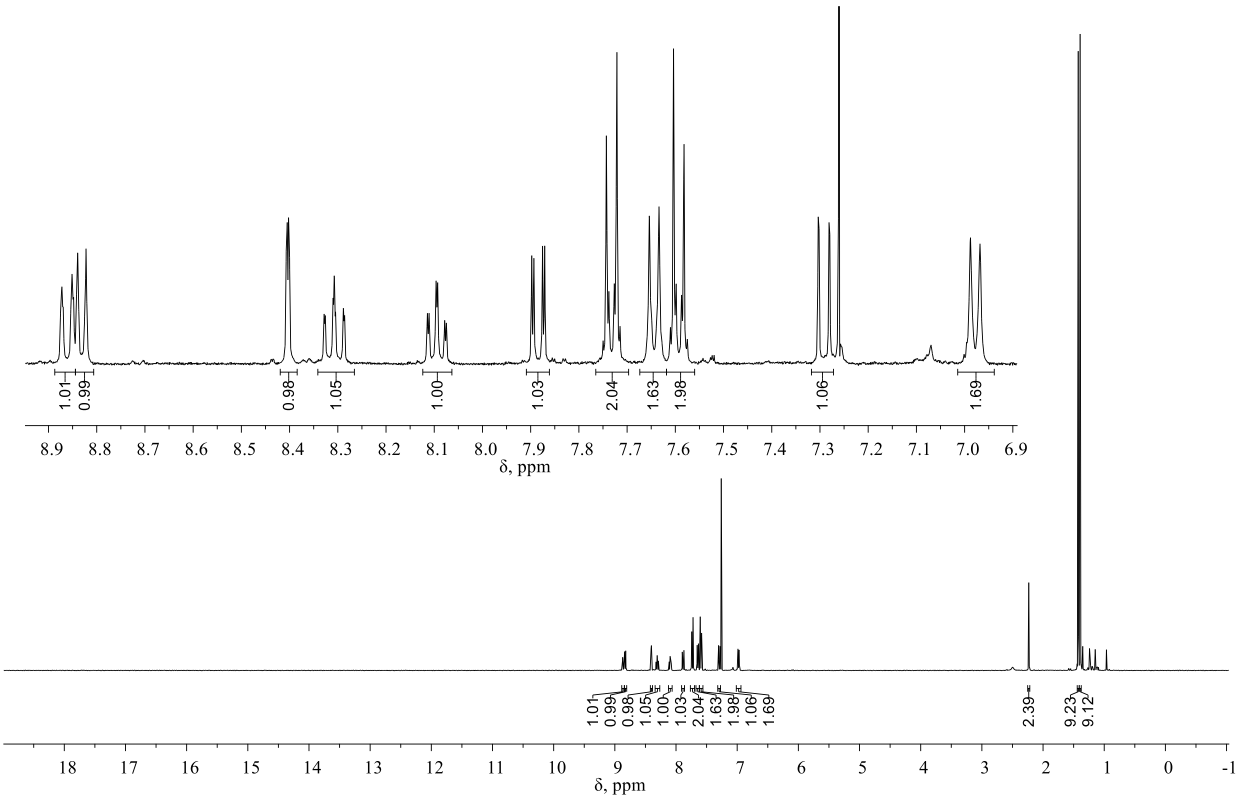


### 6.2 $^{13}$**C NMR** spectrum of **S1$^{+}$OTs$^{-}$**

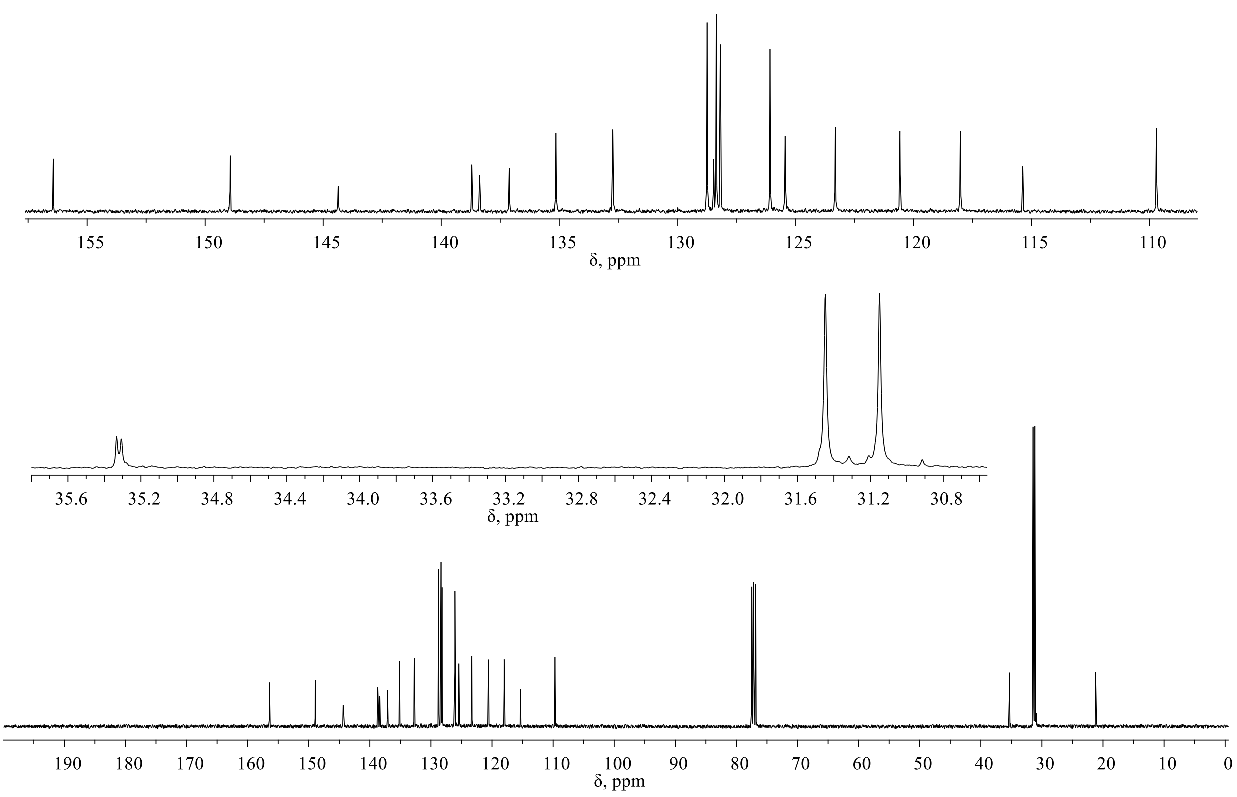

6.3 **HSQC** spectrum of **S1$^{+}$OTs$^{-}$**

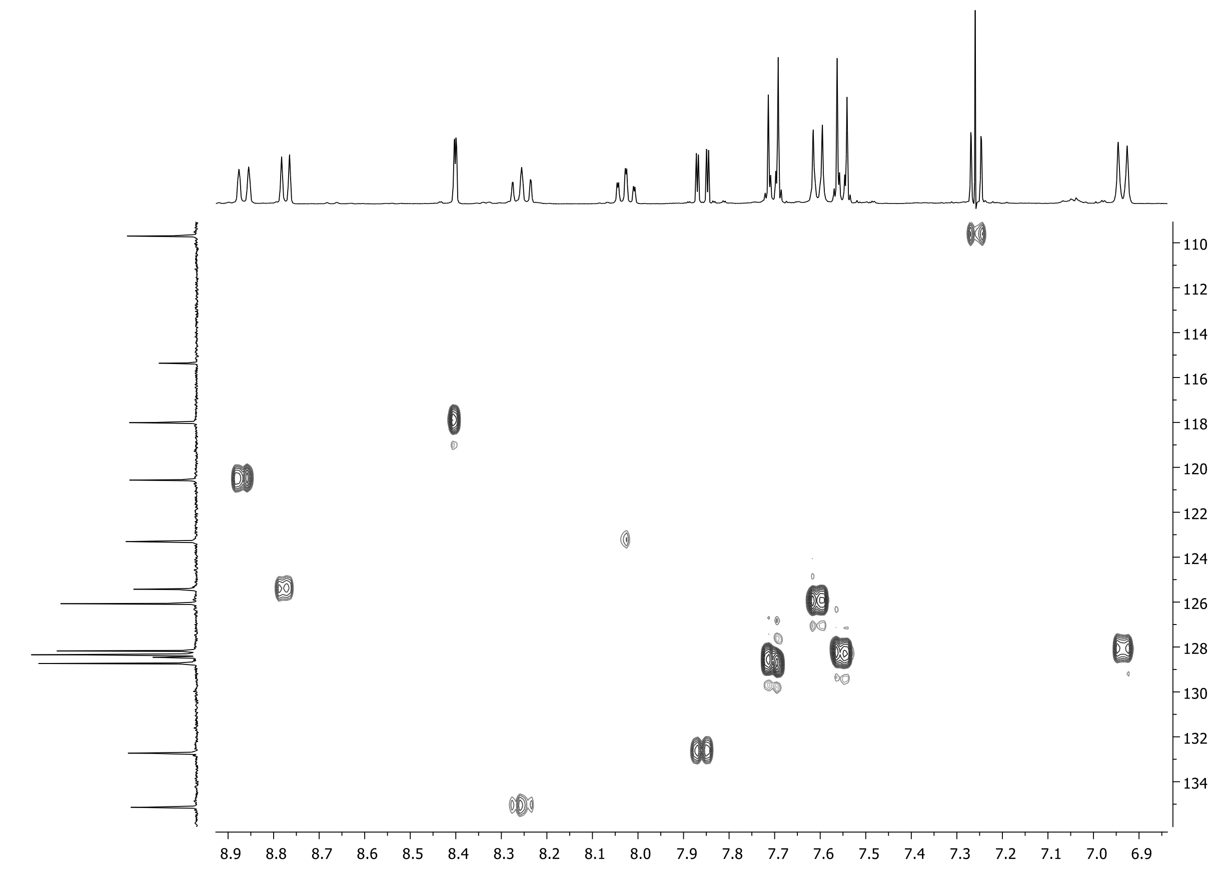

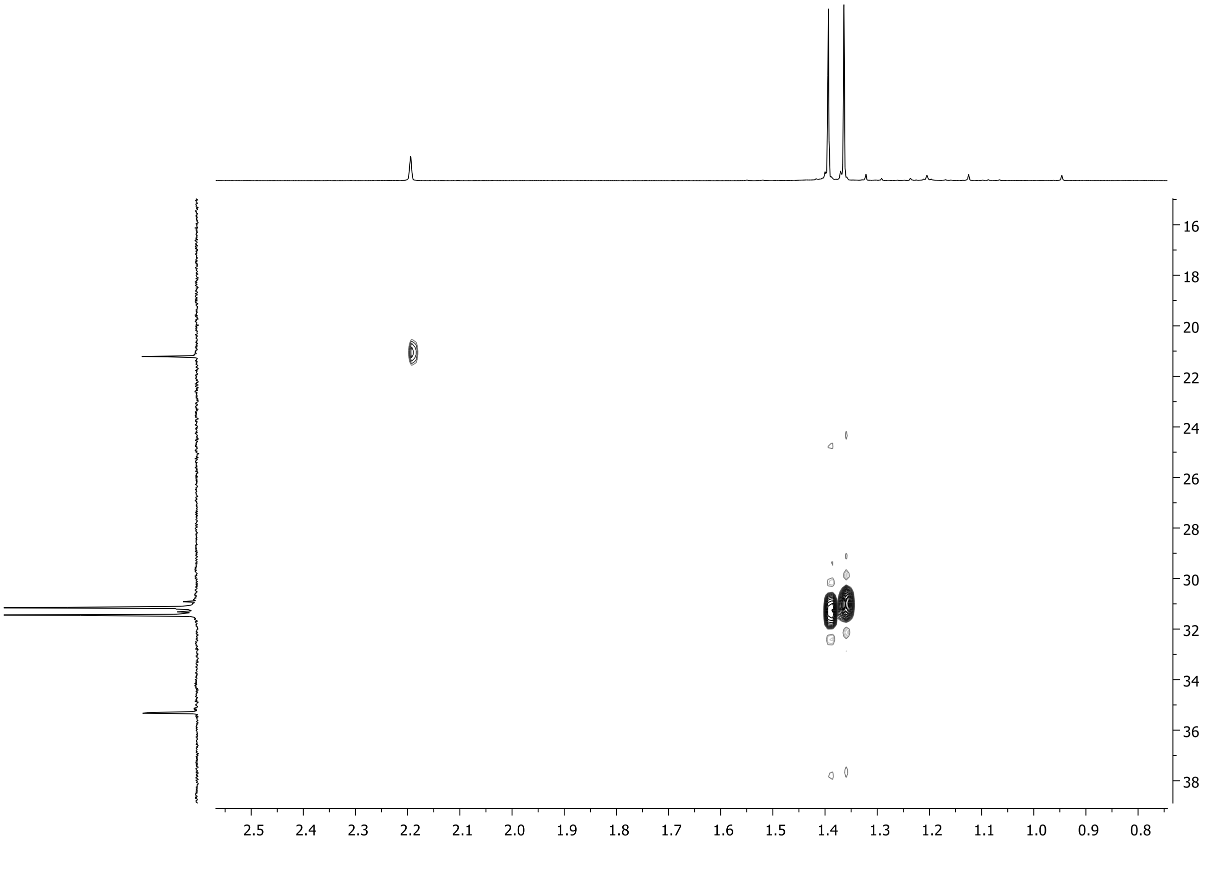

6.4 HMBC spectrum of **S1$^+$OTs$^-$**

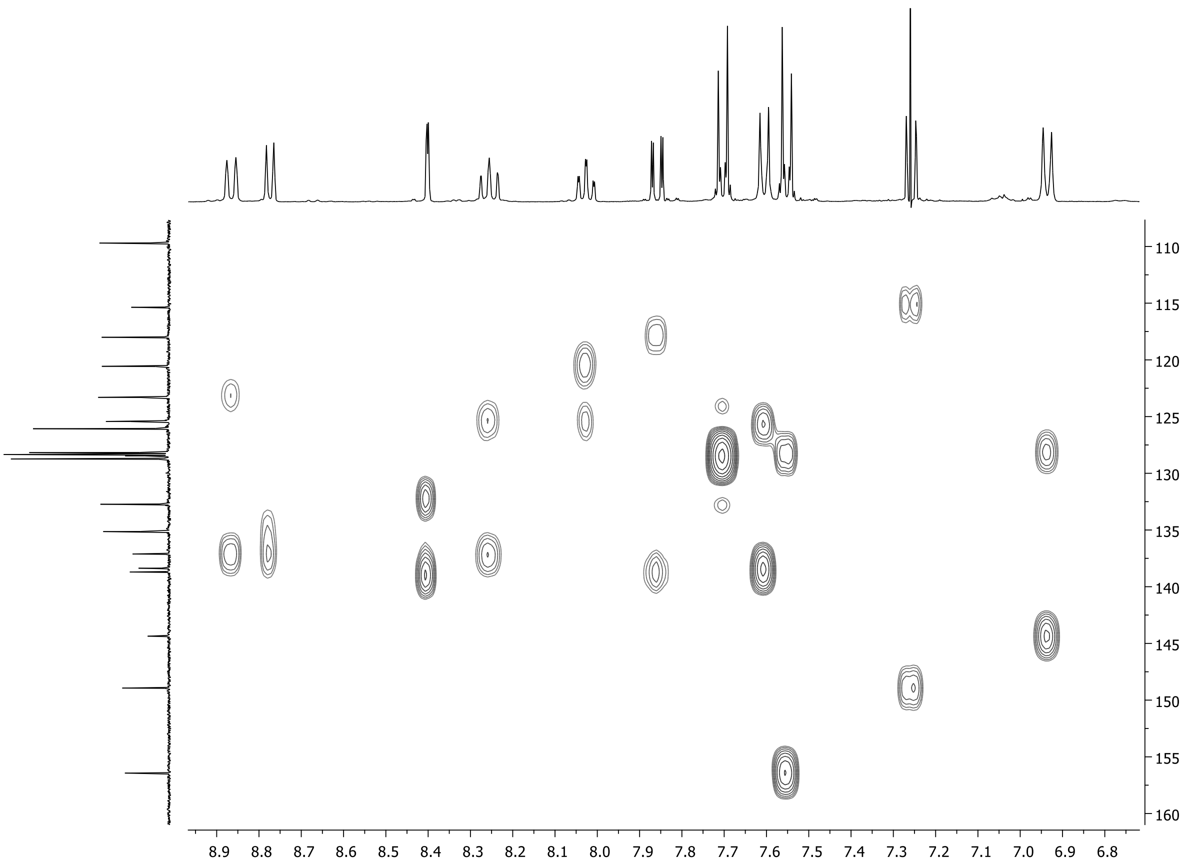


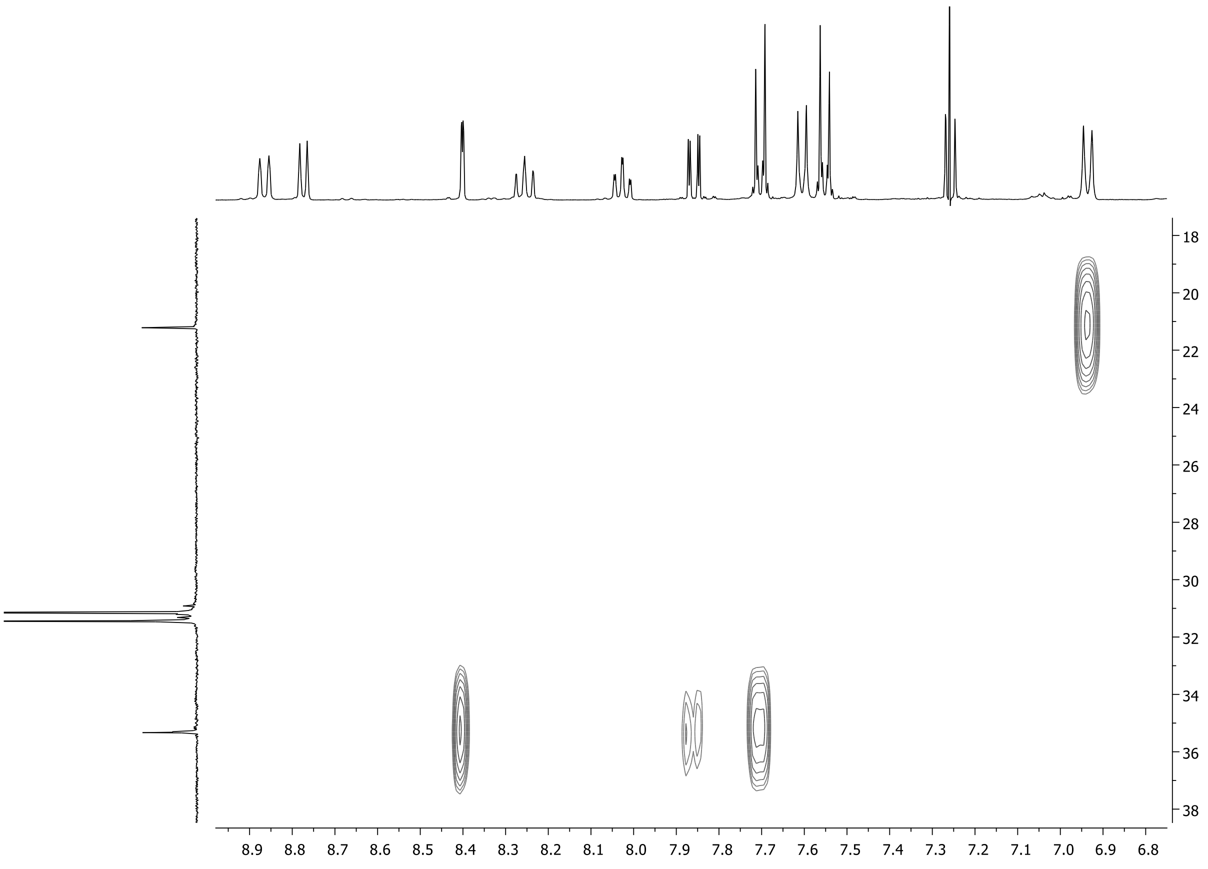

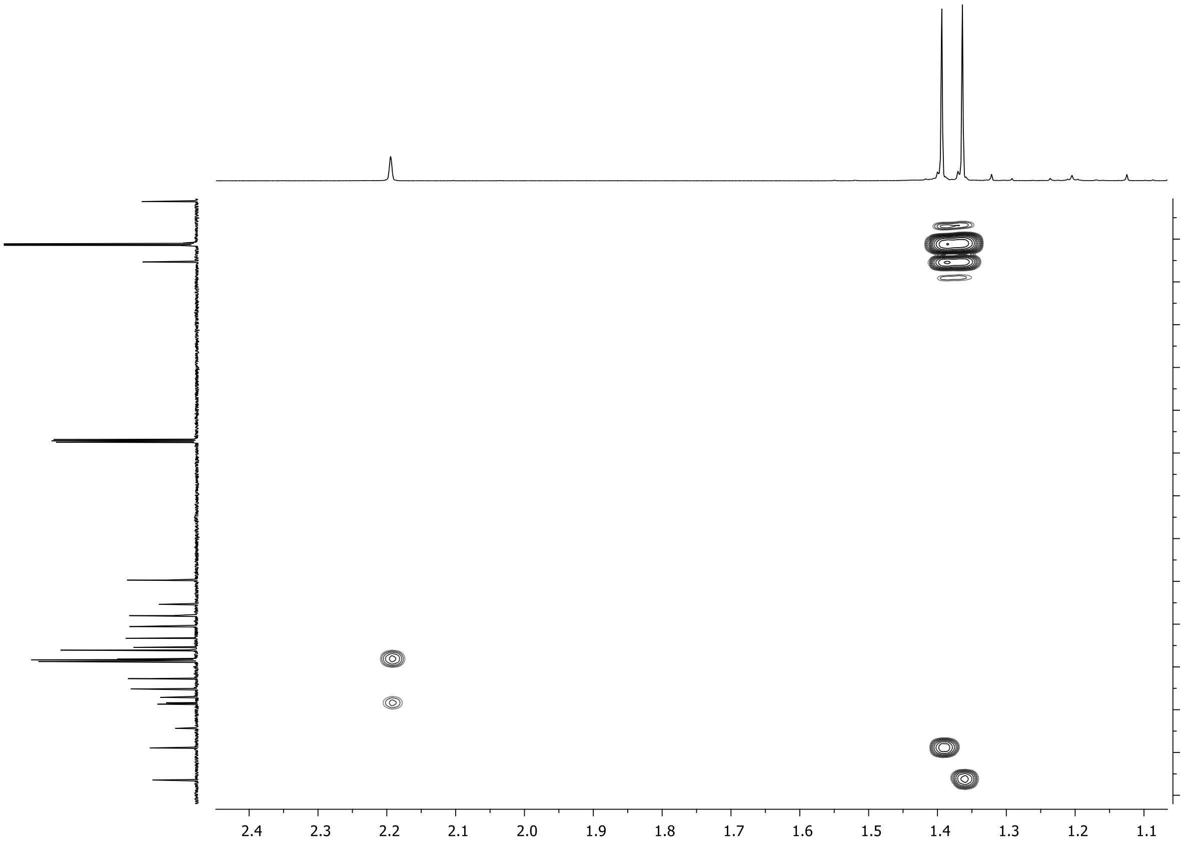

## 6.5 $^1$**H NMR** spectrum of **S1$^+$CF$_3$CO$_2$$^-$**

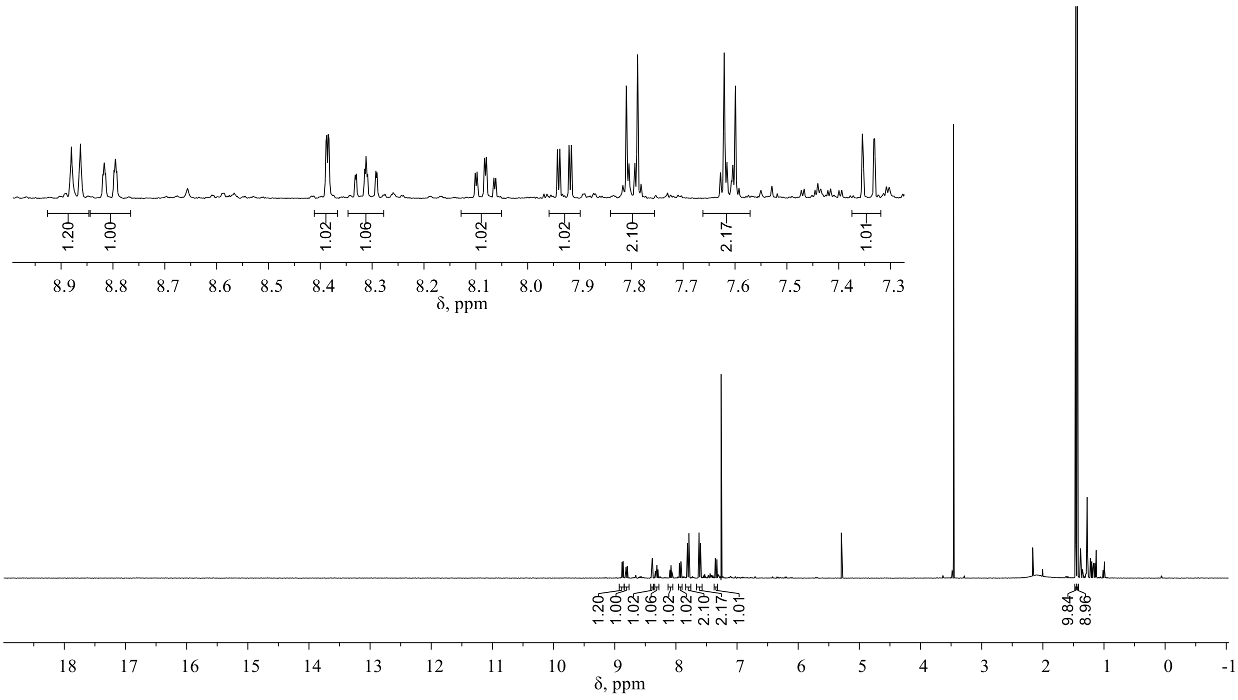

6.6 $^{1}$H NMR spectrum of $S2^{+}CF_3CO_2^{-}$

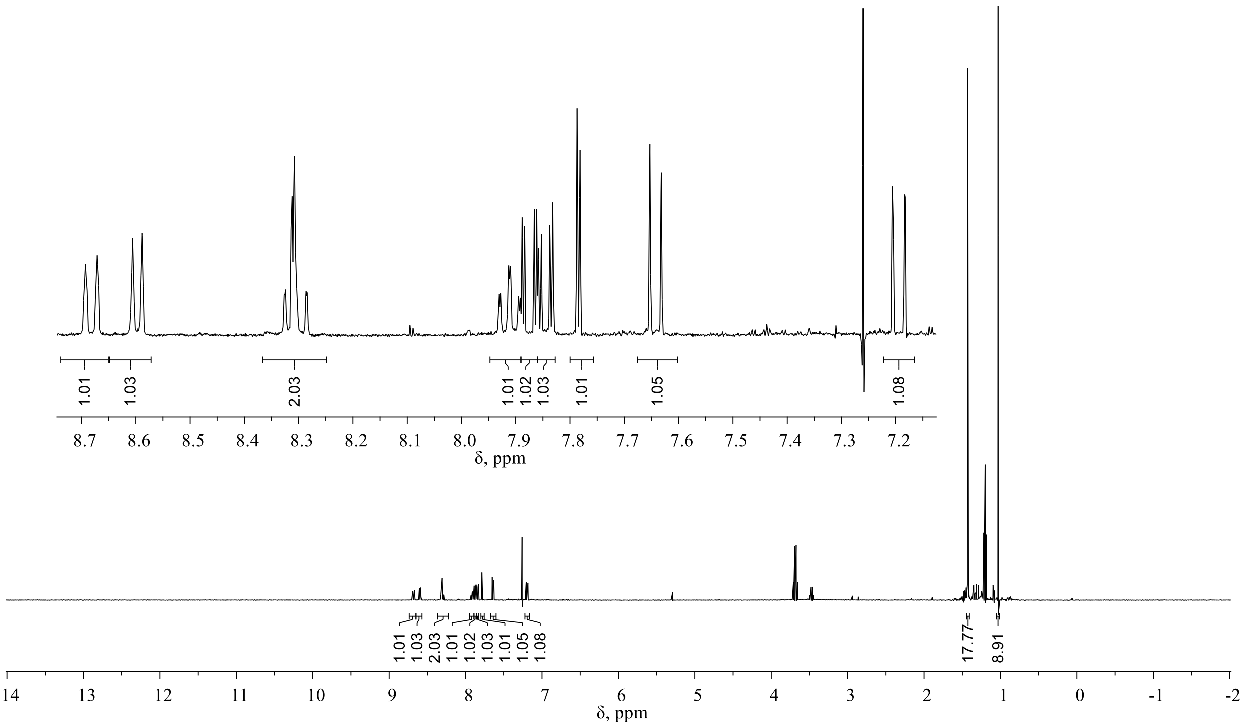


6.7 $^{13}$C NMR spectrum of $S2^{+}CF_3CO_2^{-}$

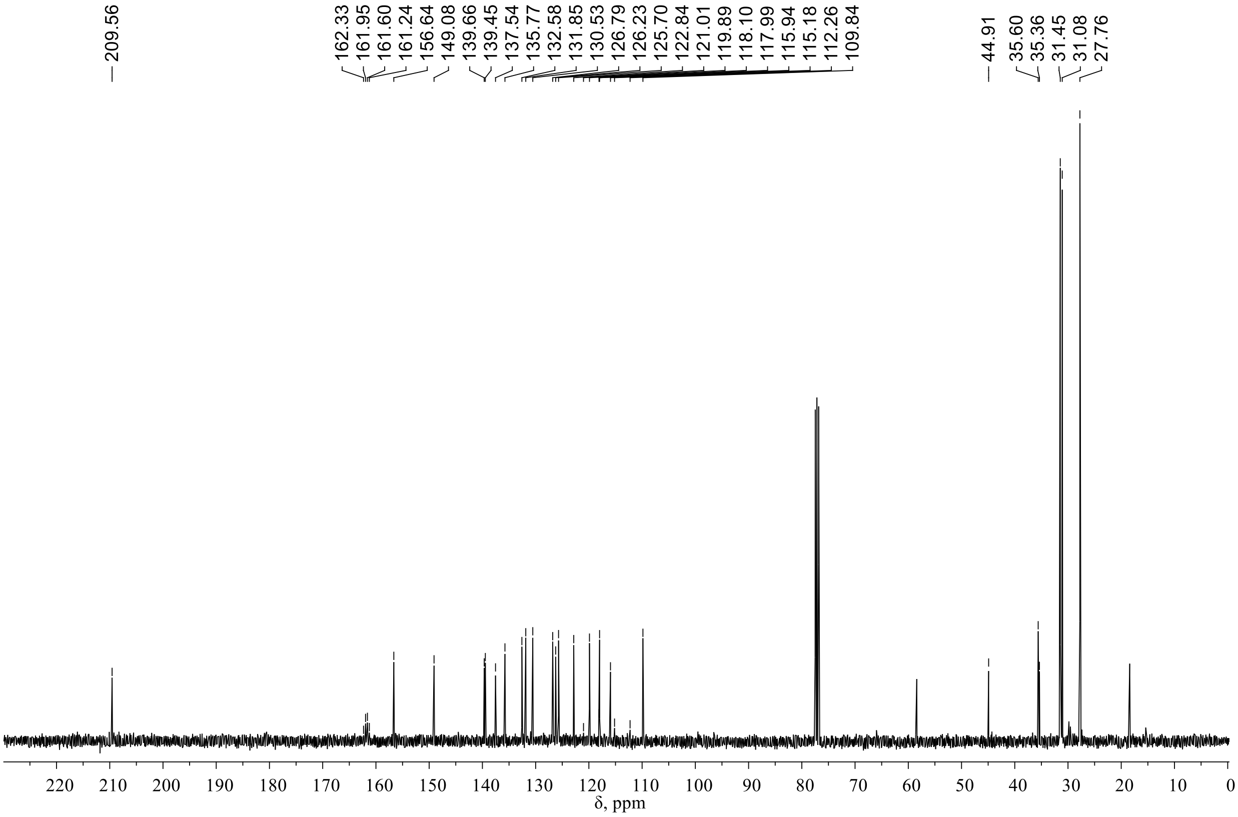

6.8 $^{19}$**F NMR** spectrum of **S2$^+$CF$_3$CO$_2$$^-$**

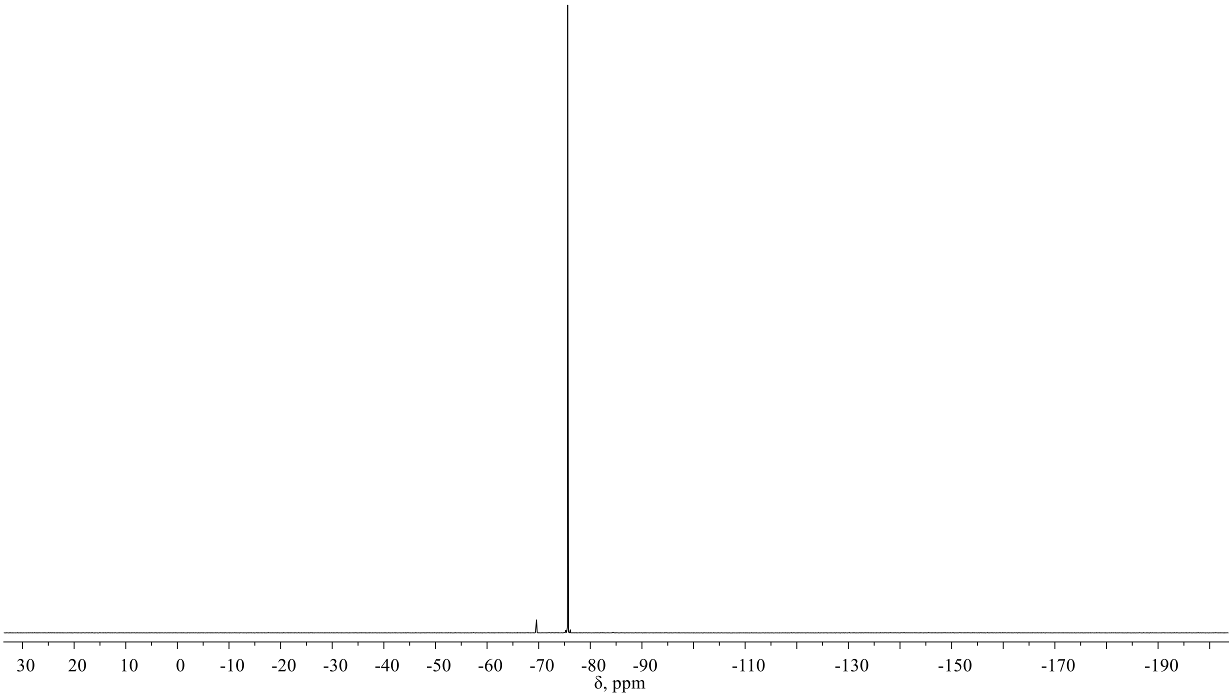


6.9 **COSY** spectrum of **S2$^+$CF$_3$CO$_2$$^-$**

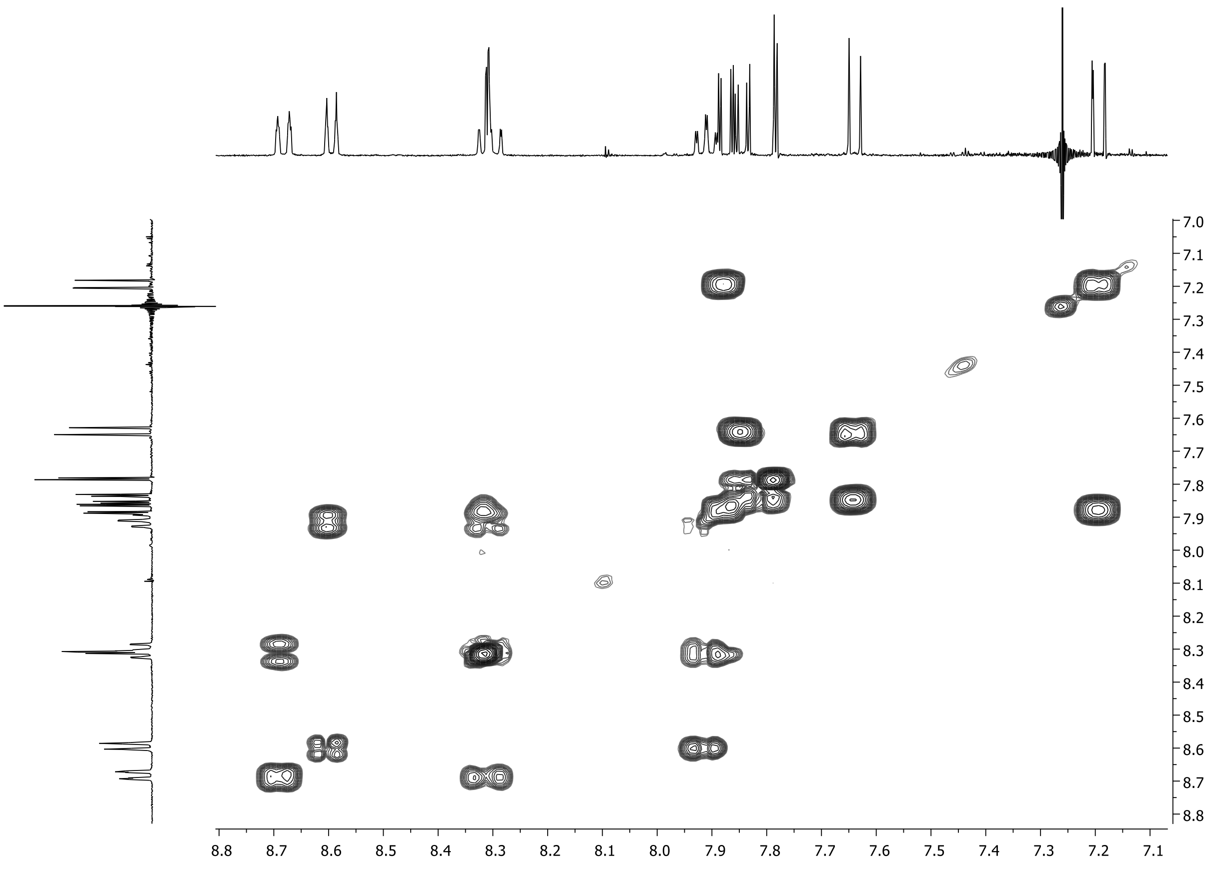

6.10 **HSQC** spectrum of **$S2^{+}CF_3CO_2^{-}$**

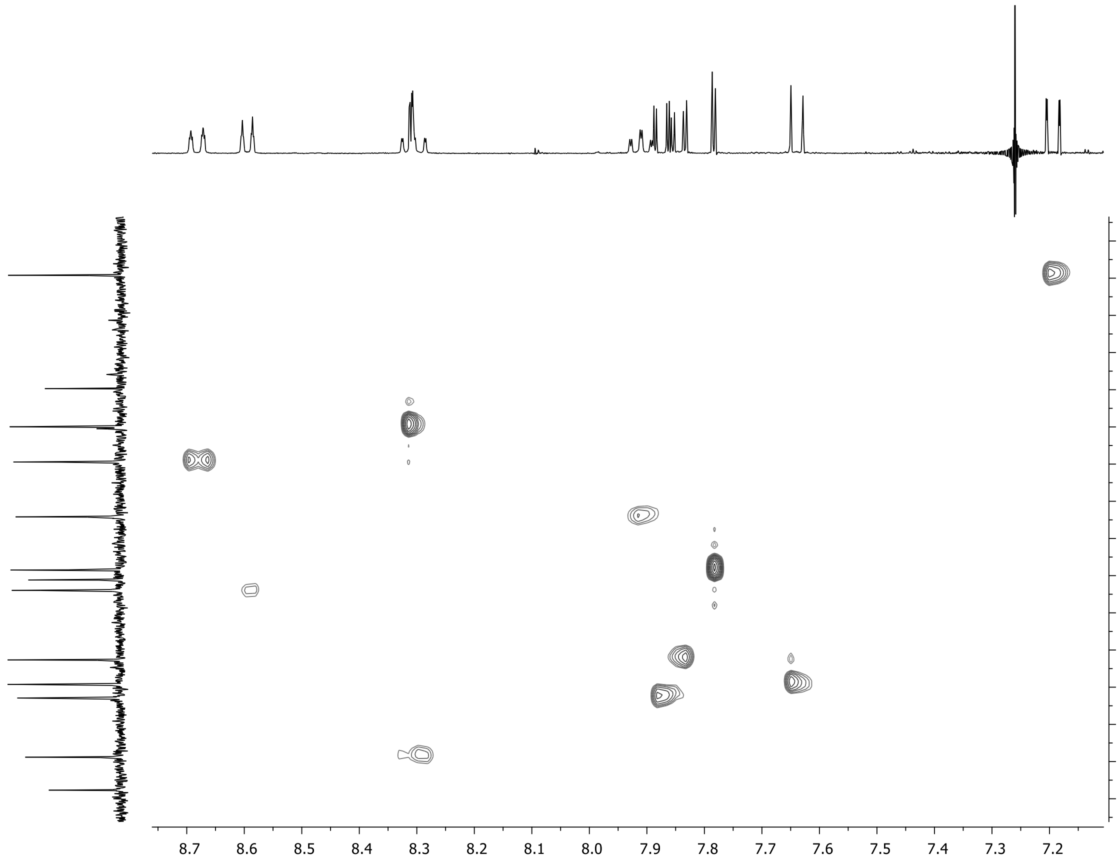


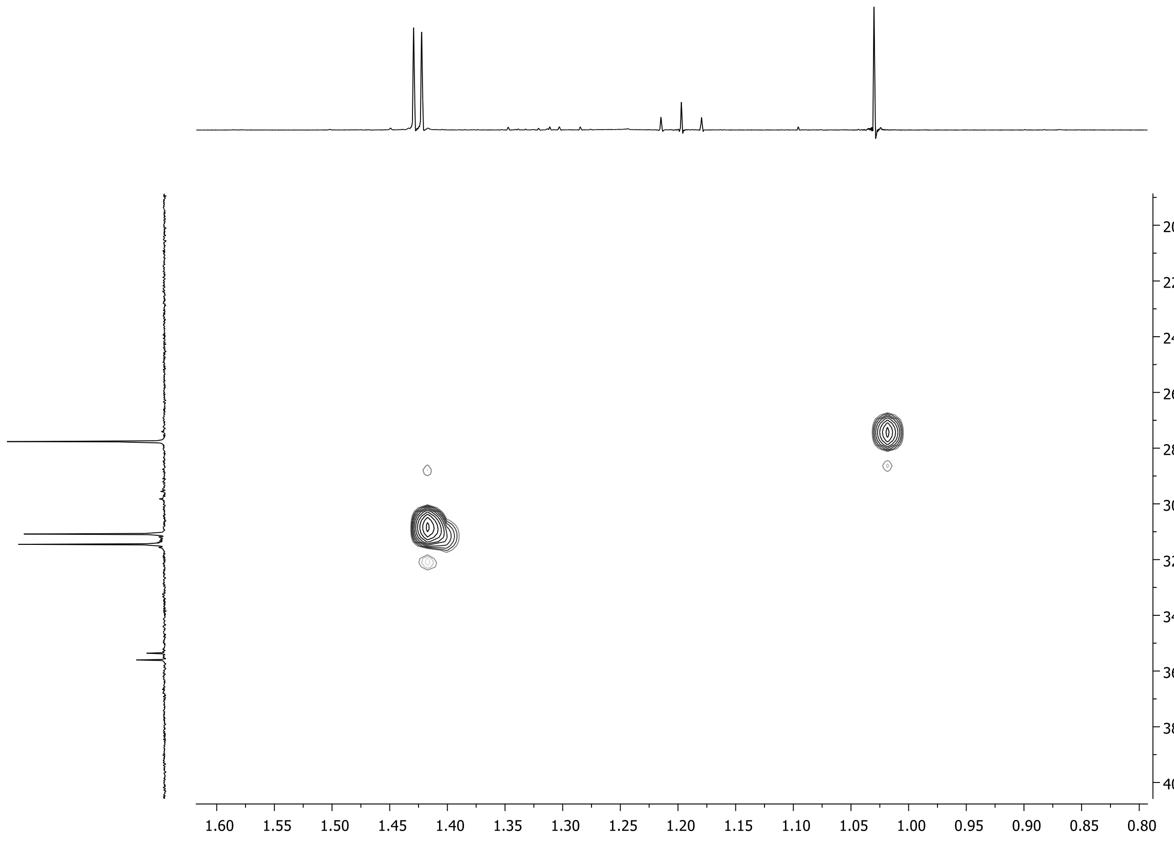

6.11 **HMBC** spectrum of **S2$^+$CF$_3$CO$_2^-$**

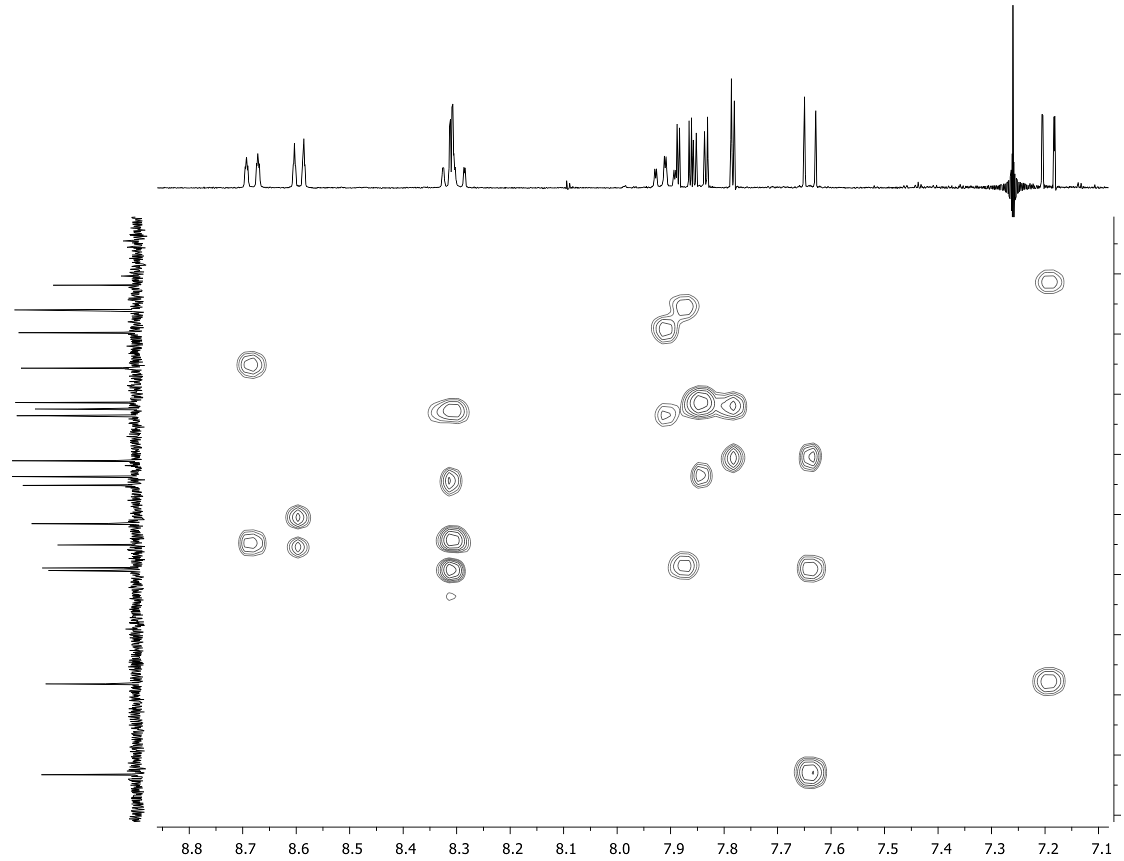


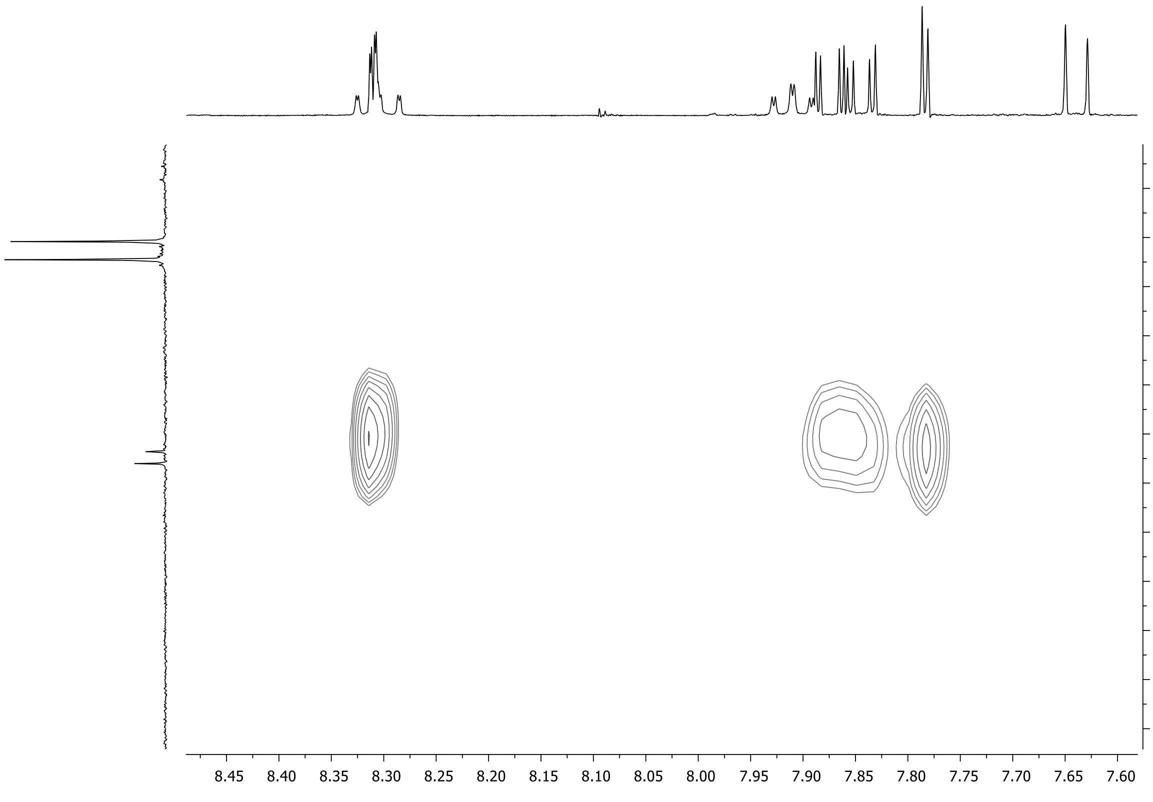

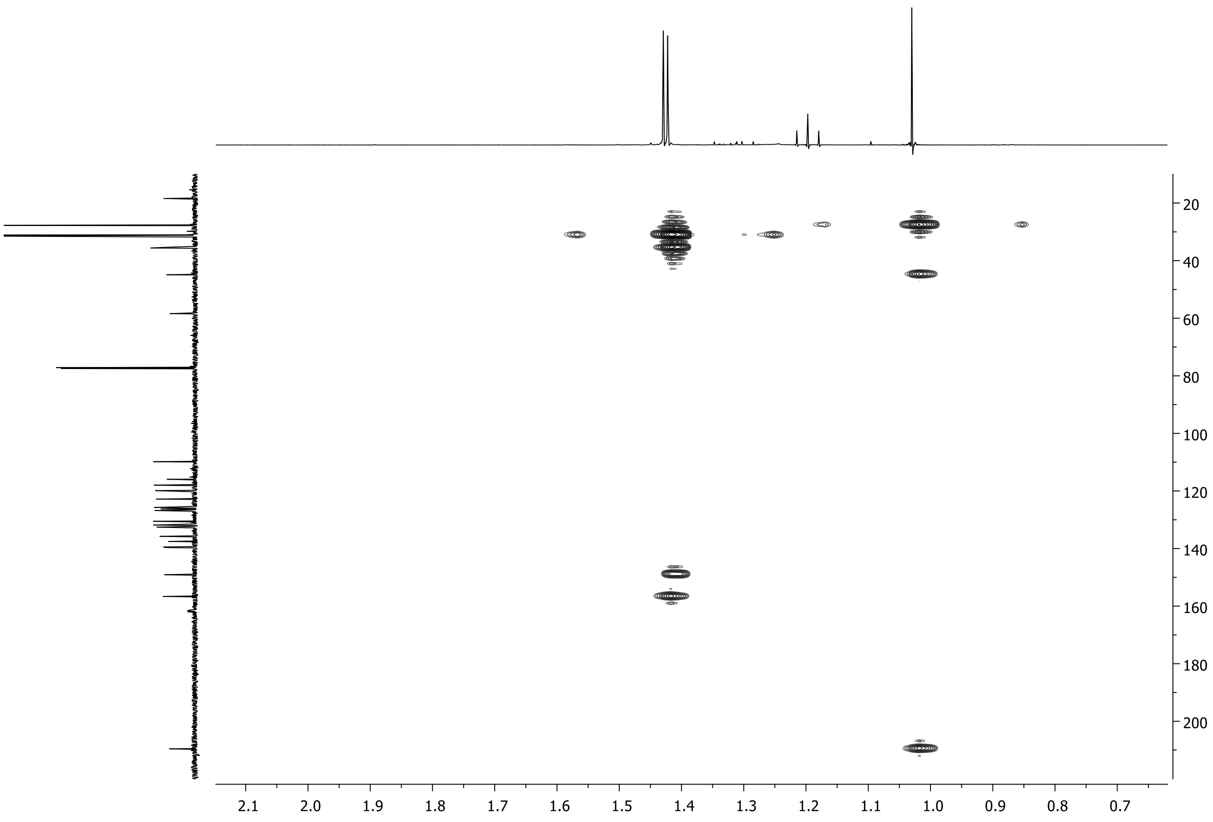

20
40
60
80
100
120
140
160
180
200
2.1
2.0
1.9
1.8
1.7
1.6
1.5
1.4
1.3
1.2
1.1
1.0
0.9
0.8
0.7


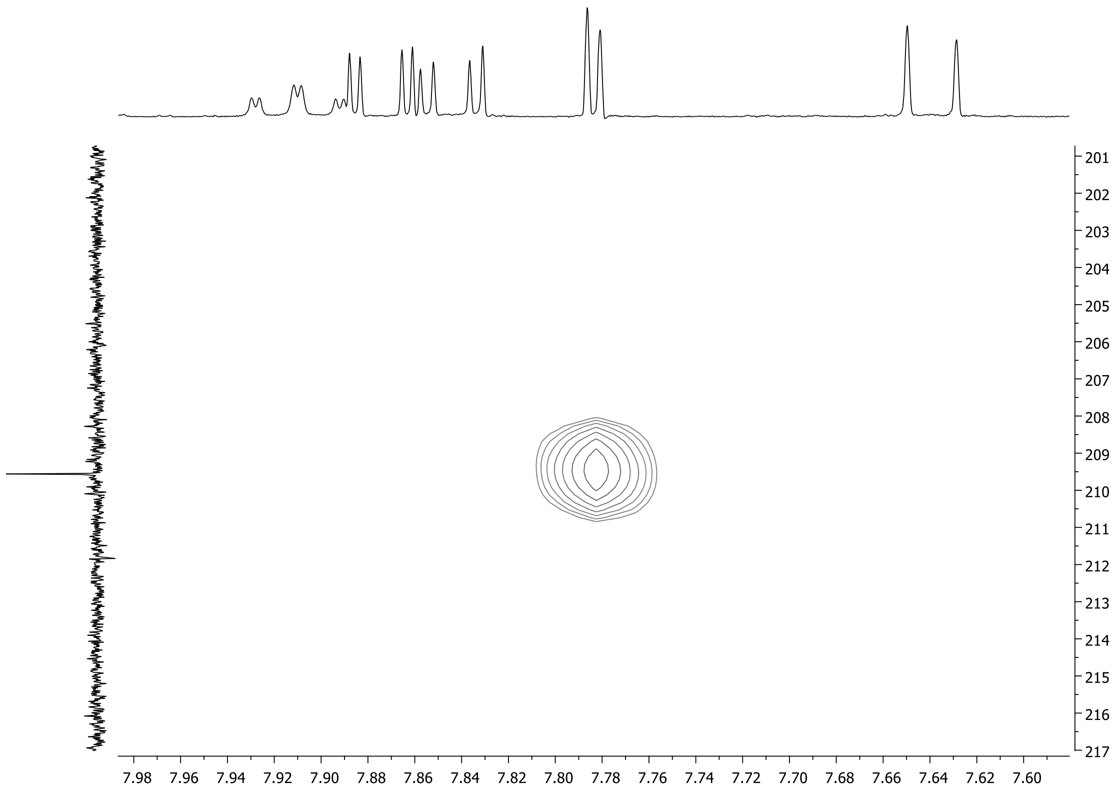

201
202
203
204
205
206
207
208
209
210
211
212
213
214
215
216
217
7.98
7.96
7.94
7.92
7.90
7.88
7.86
7.84
7.82
7.80
7.78
7.76
7.74
7.72
7.70
7.68
7.66
7.64
7.62
7.60

6.12 $^{1}$**H NMR** spectrum of **S2$^{+}$OTs$^{-}$**

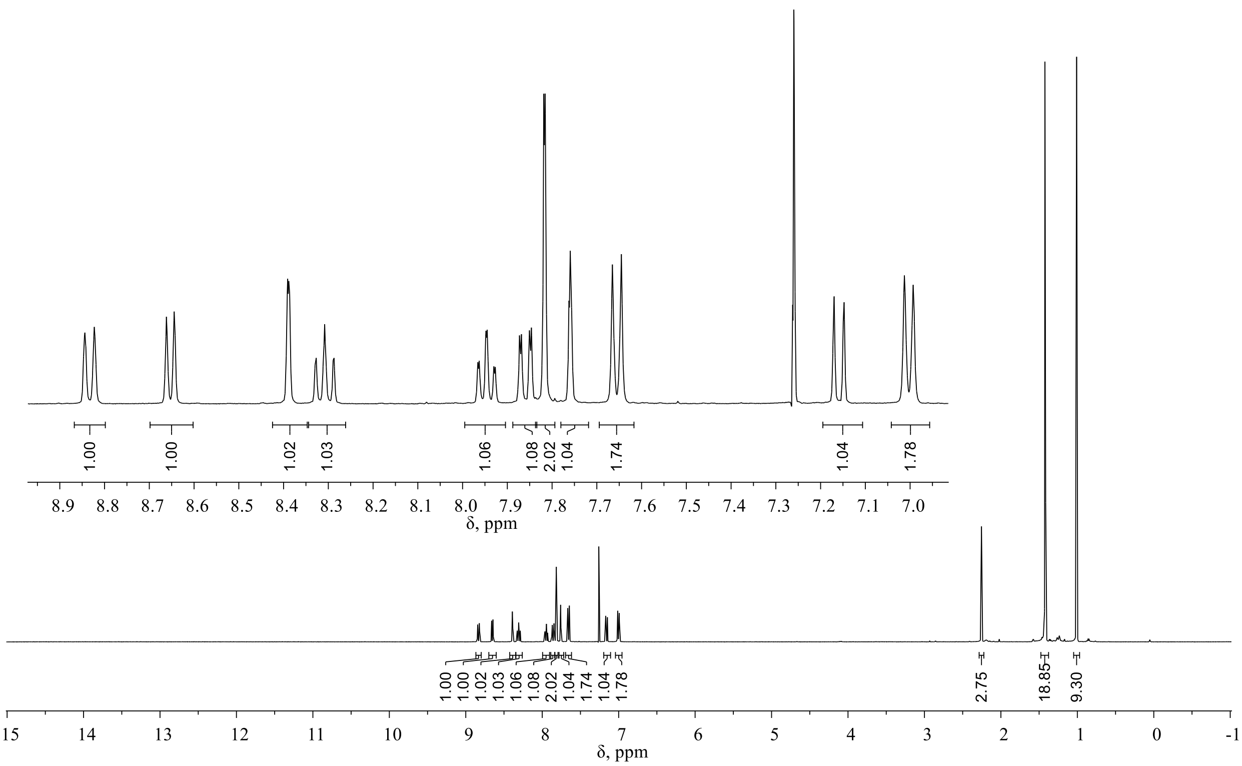


6.13 $^{13}$**C NMR** spectrum of **S2$^{+}$OTs$^{-}$**

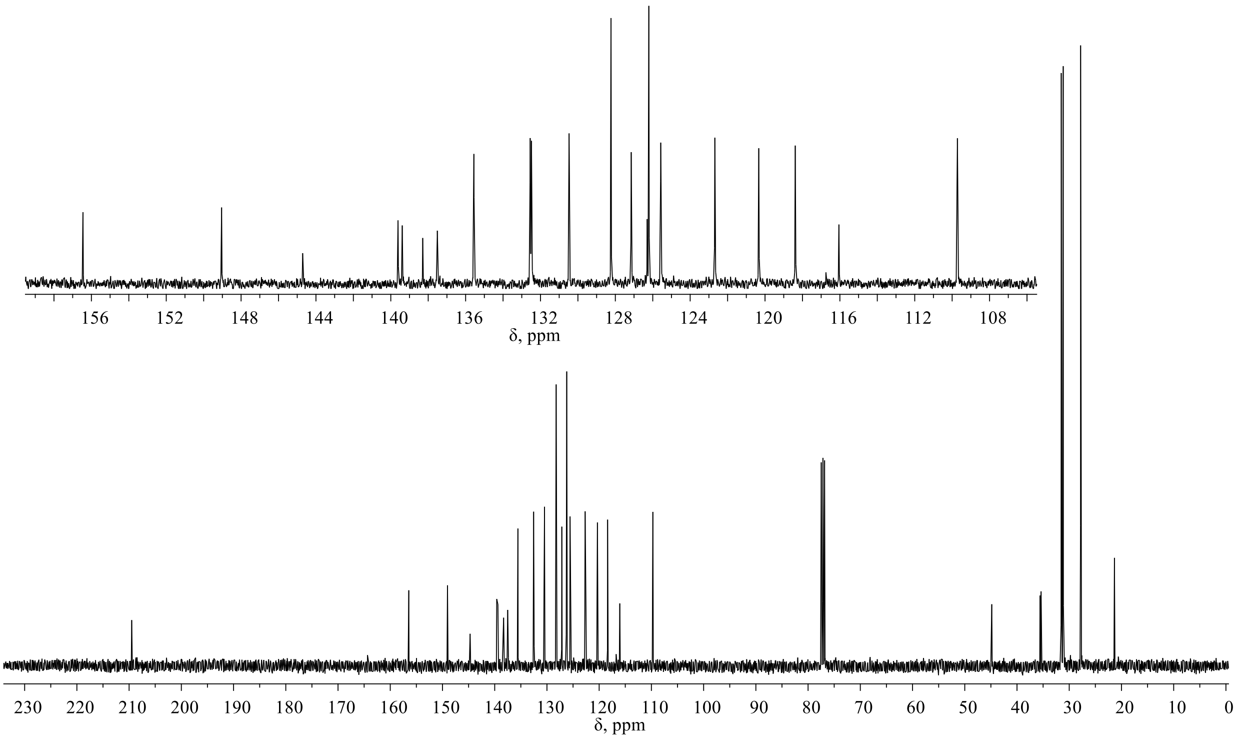

6.14 $^{1}$**H NMR** spectrum of **S3$^{+}$CF$_3$CO$_2^{-}$**

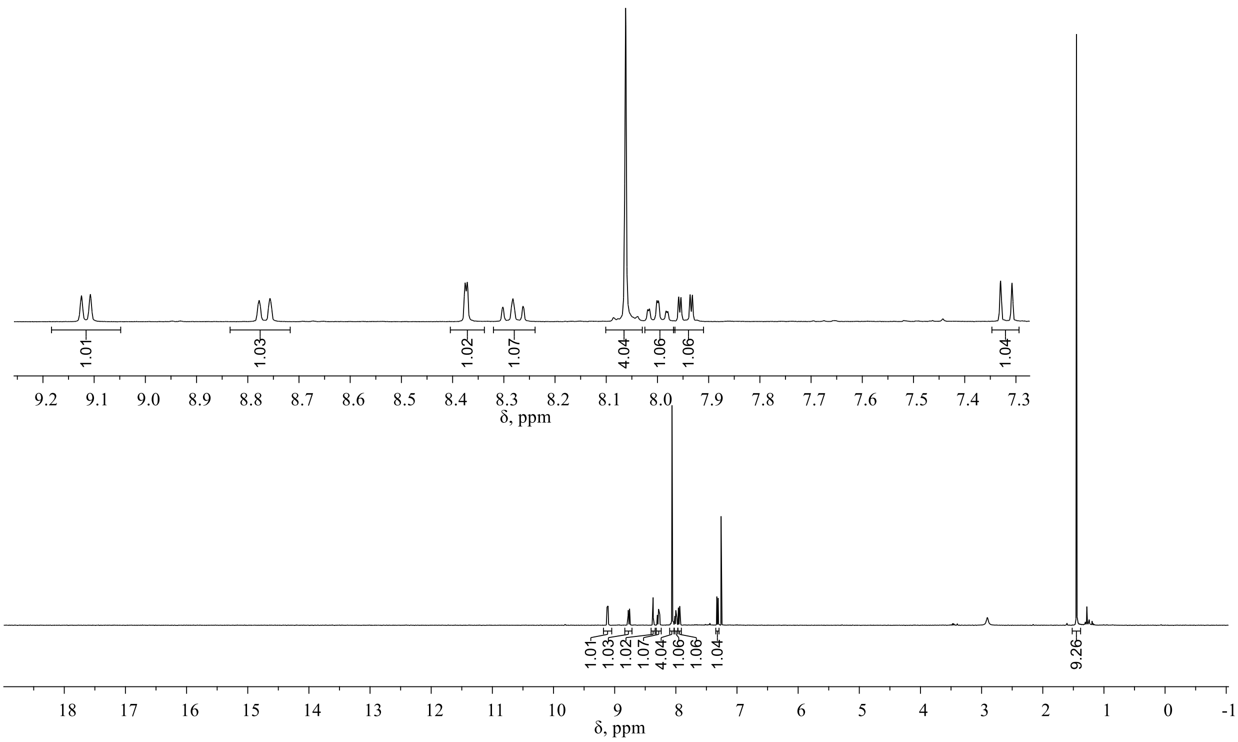

6.15 $^{13}$**C NMR** spectrum of **S3$^{+}$CF$_3$CO$_2^{-}$**

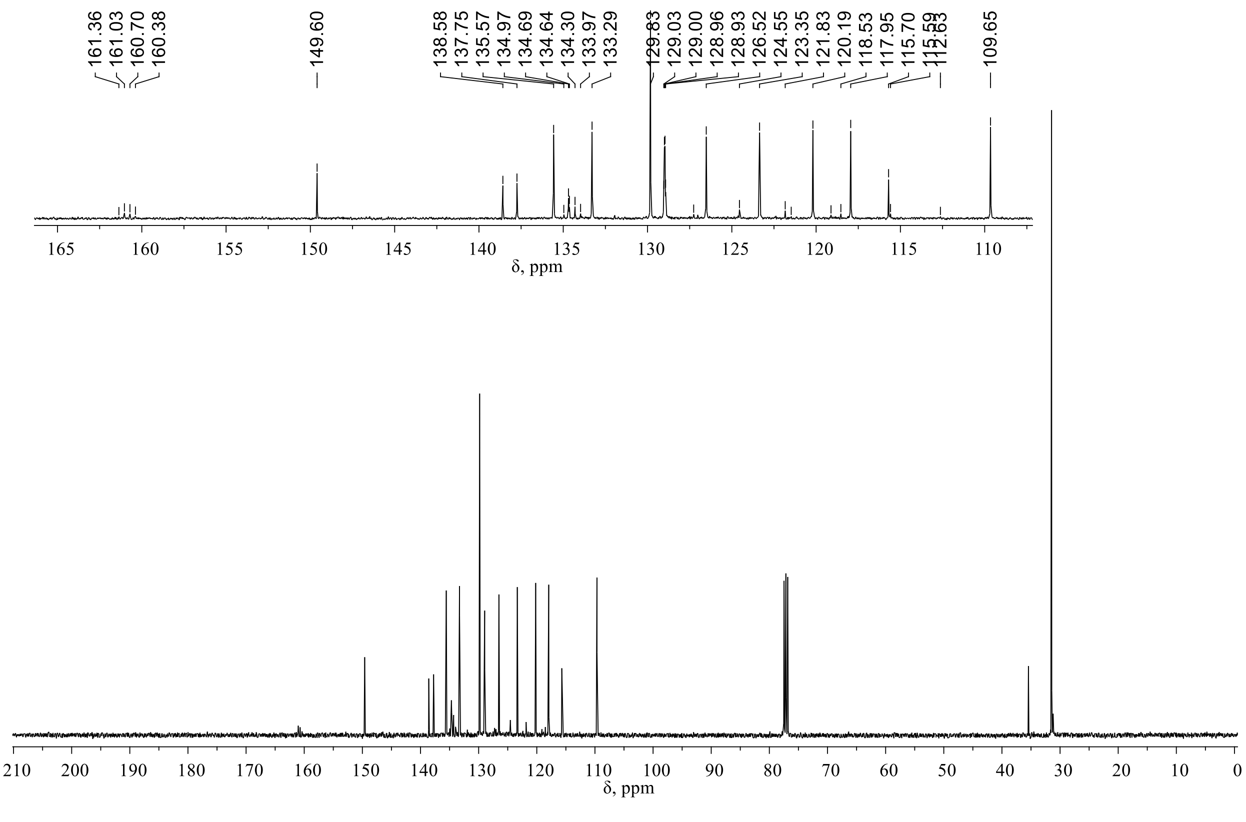

6.16 **$^{19}$F NMR** spectrum of **$S3^{+}CF_3CO_2^{-}$**

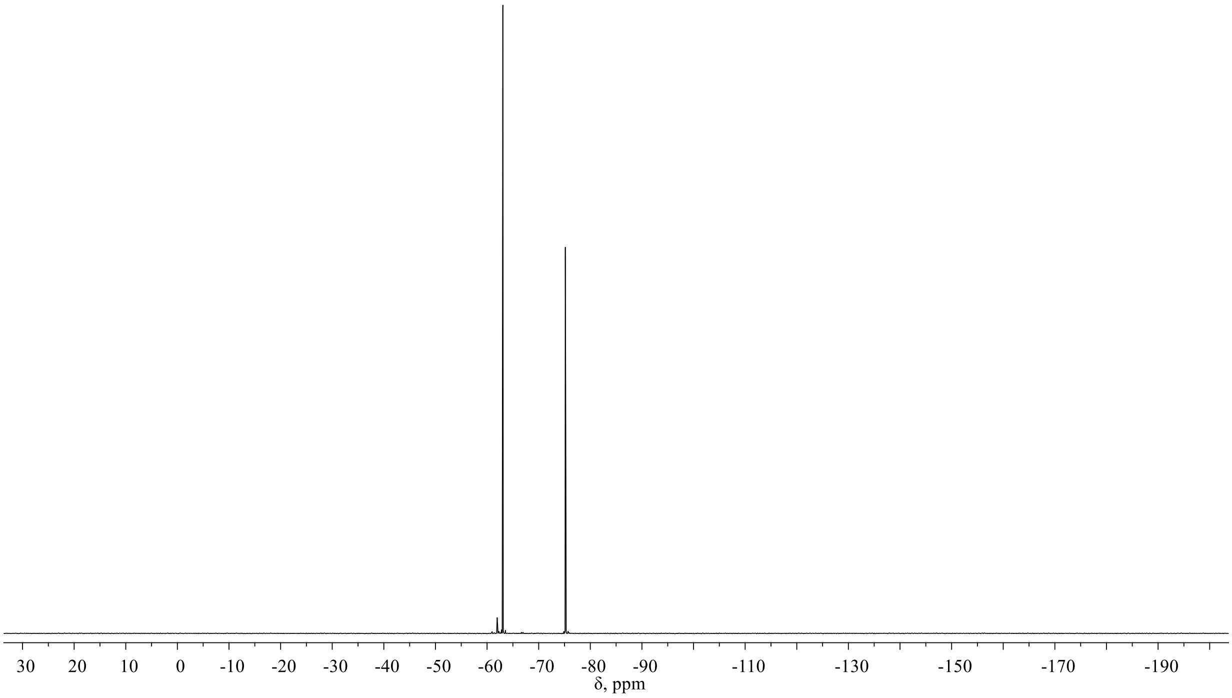


6.17 **HSQC** spectrum of **$S3^{+}CF_3CO_2^{-}$**

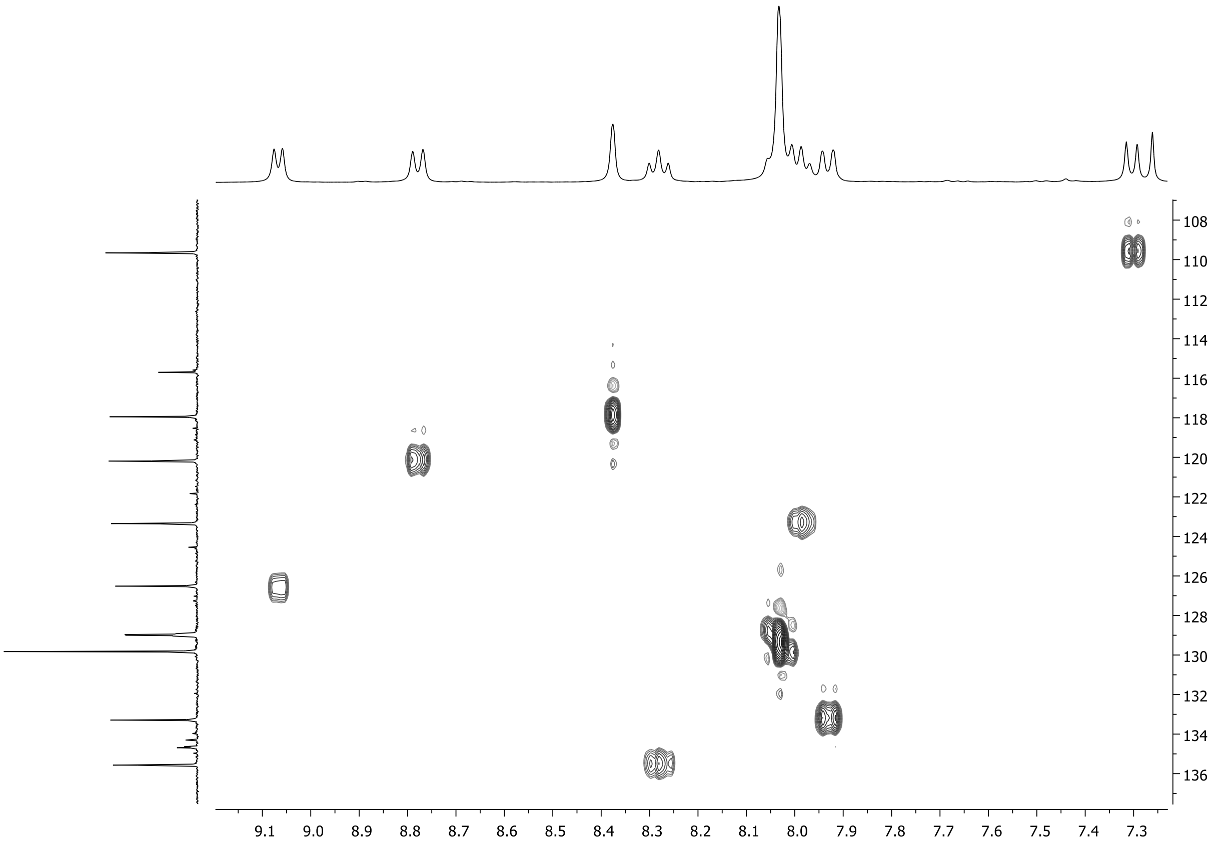

6.18 **HMBC** spectrum of **S3**$^{+}$**$CF_3CO_2$**$^{-}$

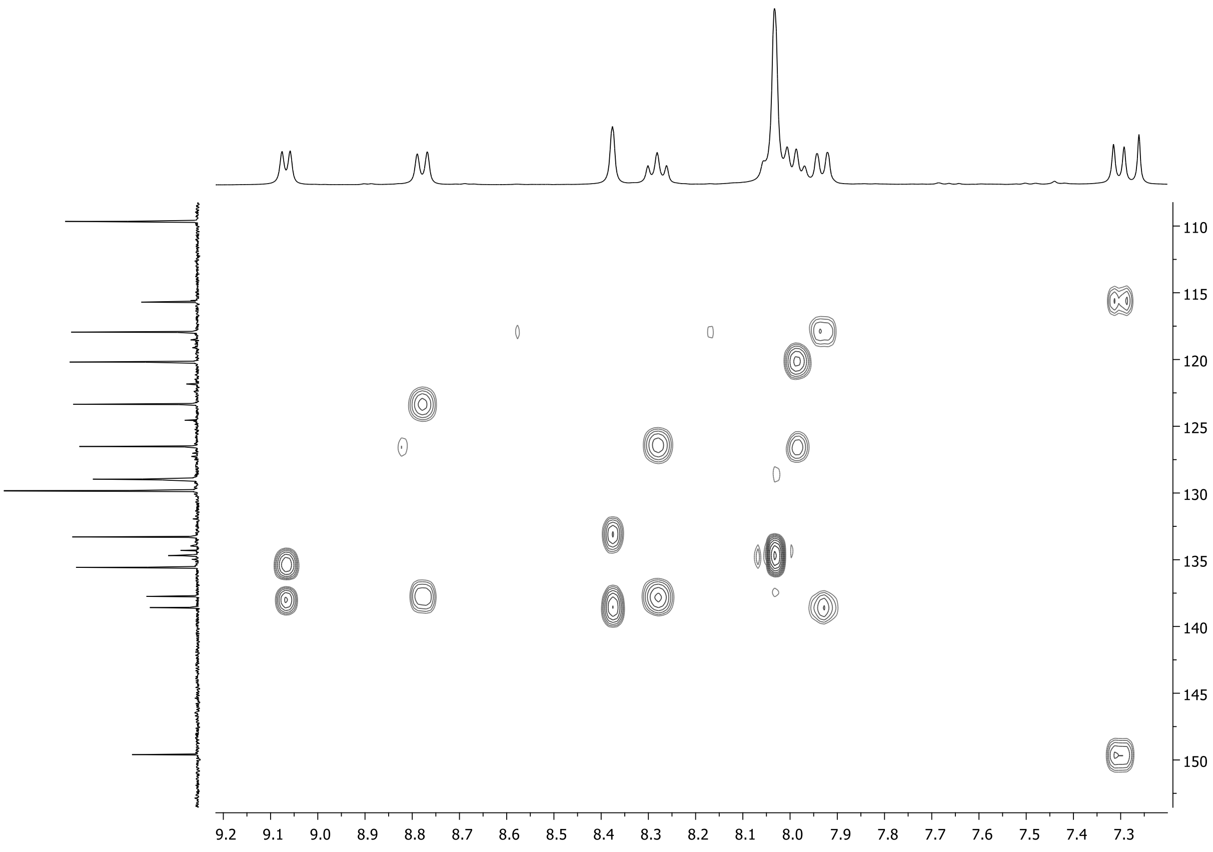


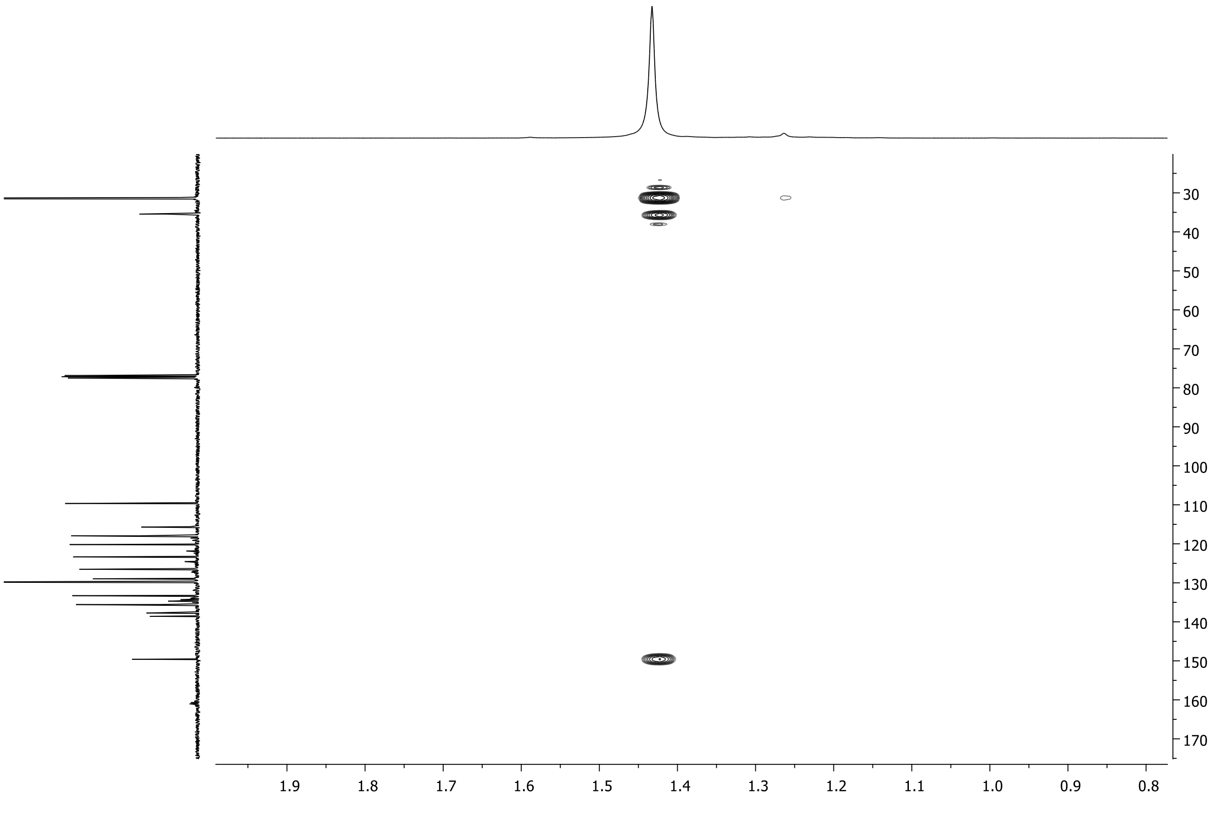

6.19 **¹H NMR** spectrum of a mixture of amine **A3** and its dimer (tetraarylhydrazine) obtained from the TEMPO-mediated electrolysis

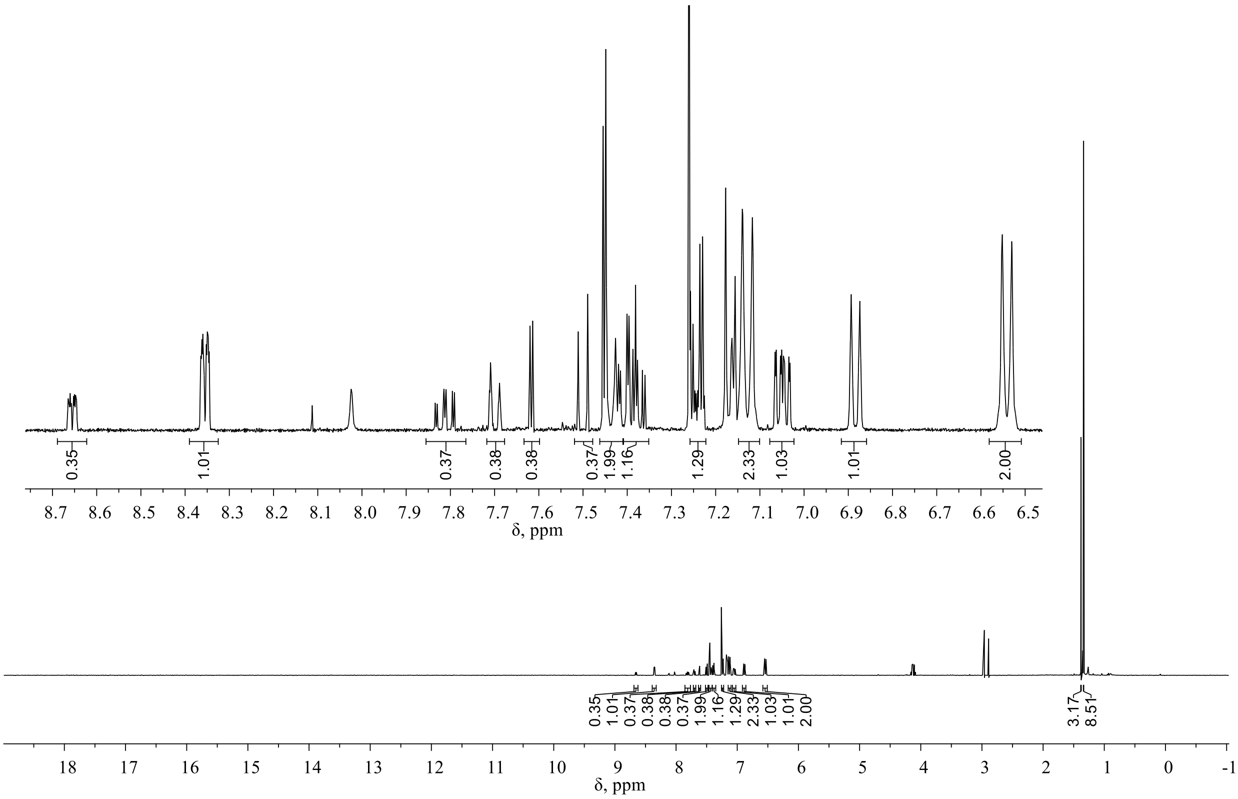


6.20 **¹H NMR** spectrum of **S3⁺OTs⁻** obtained from the TEMPO-mediated electrosynthesis

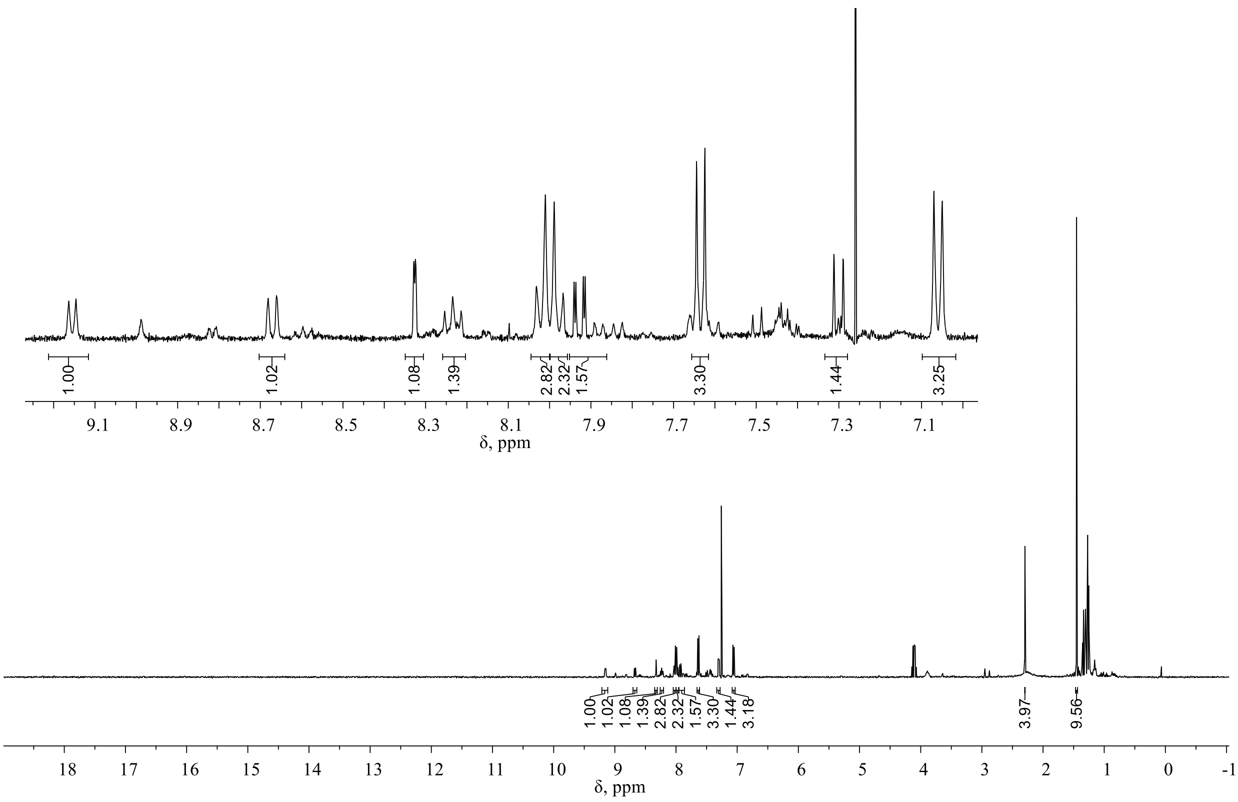

6.21 **GC-MS** analysis results for a mixture of amine **A3** and its dimer (tetraarylhydrazine) obtained from the TEMPO-mediated electrolysis

Chromatogram:

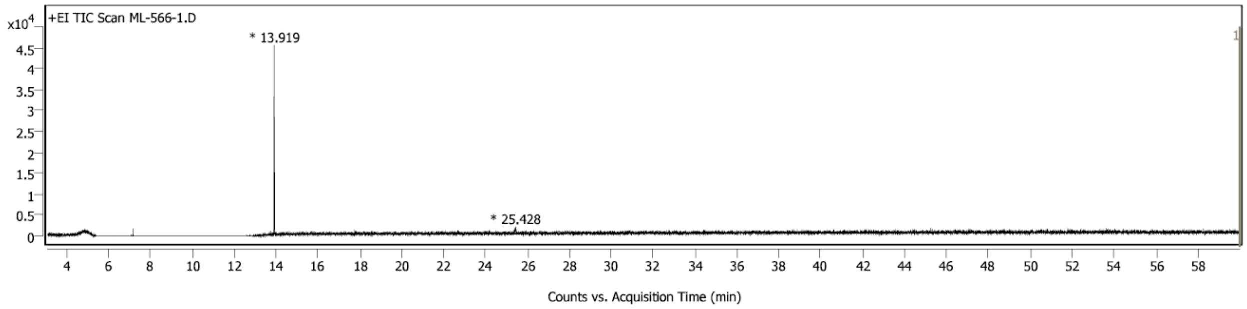


*Chromatogram Peaks*

| Peak | Start | RT | End | Height | Area | Area % | SNR |
|---|---|---|---|---|---|---|---|
| 1 | 13.807 | 13.919 | 14.012 | 45065 | 41898 | 100.00 | |
| 2 | 25.259 | 25.428 | 25.639 | 1639 | 7301 | 17.43 | |

Mass-spectrum corresponding to peak at 13.9 min (amine **A3**)

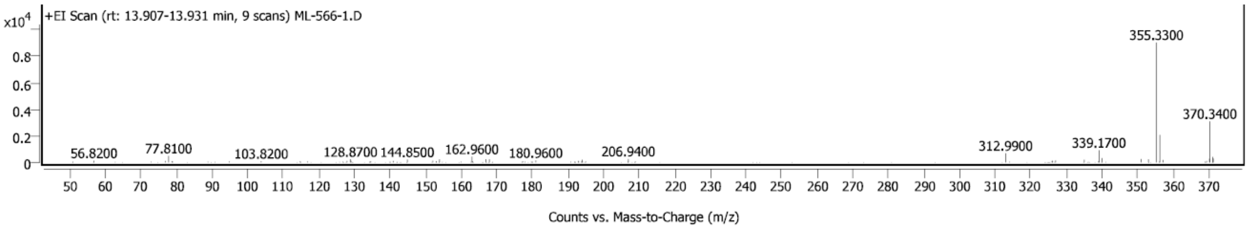


Mass-spectrum corresponding to peak at 25.4 min (N-N type dimer of amine **A3**)

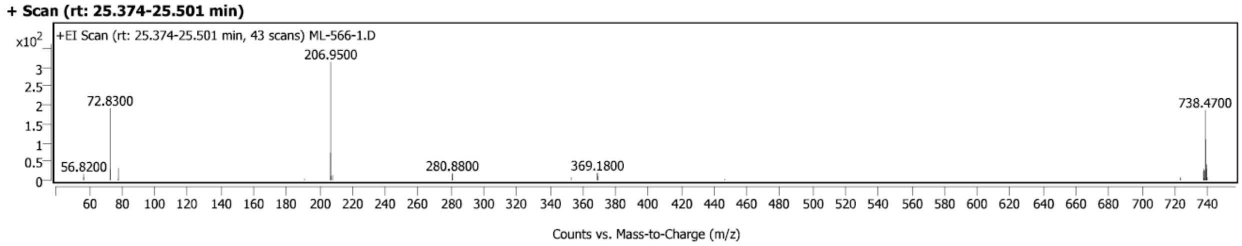


6.22 **ESI-HRMS** spectrum of **S1**$^{+}$**CF**$_3$**CO**$_2$$^{-}$

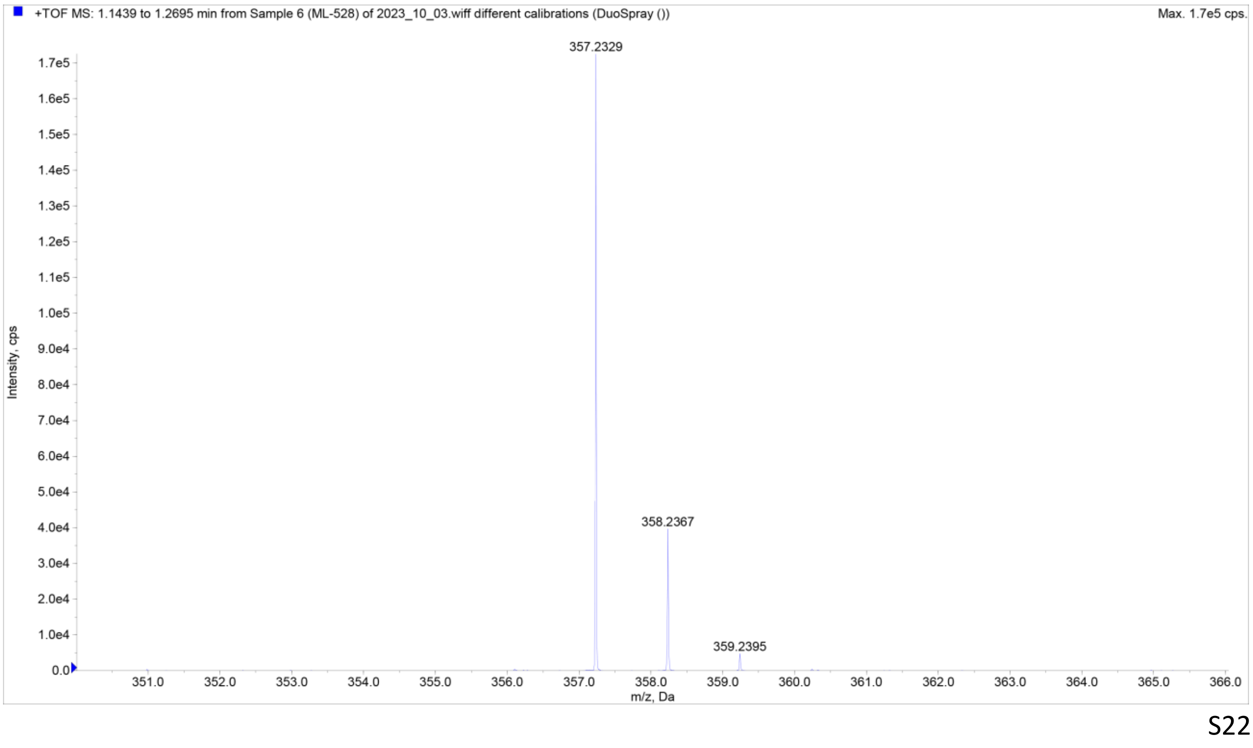

6.23 **ESI-HRMS** spectrum of **S2$^+$CF$_3$CO$_2$$^-$**

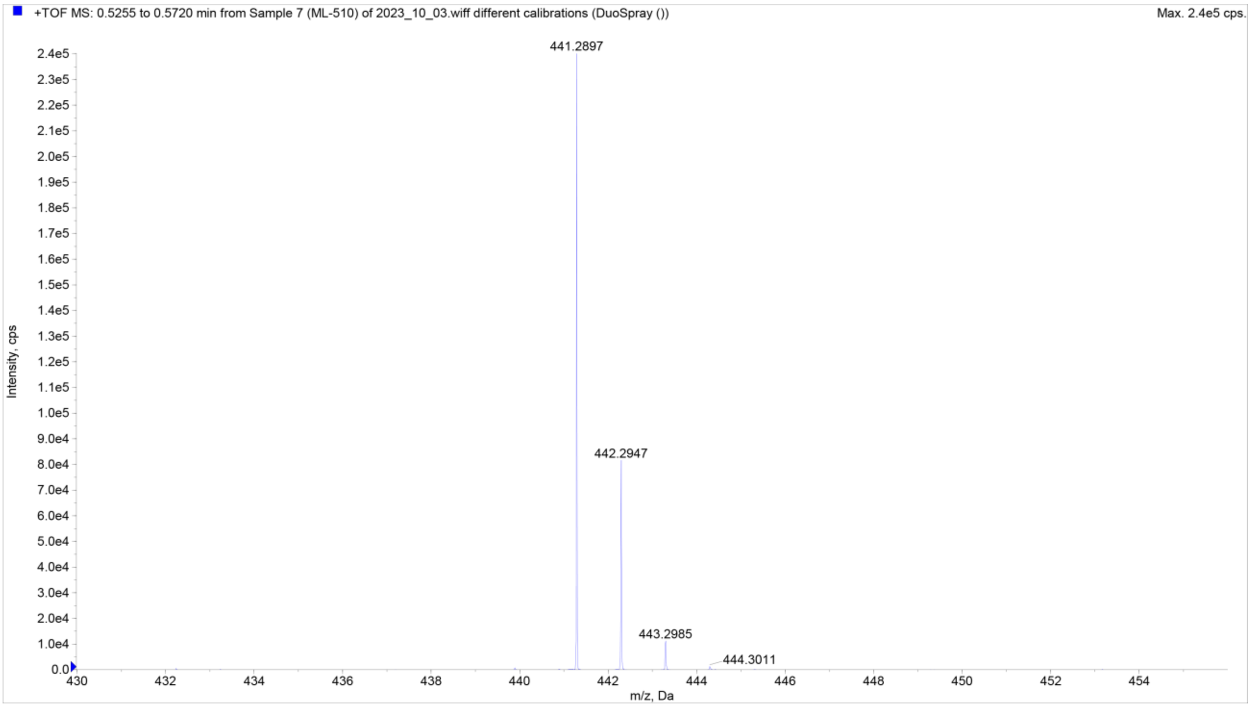


6.24 **ESI-HRMS** spectrum of **S3$^+$CF$_3$CO$_2$$^-$**

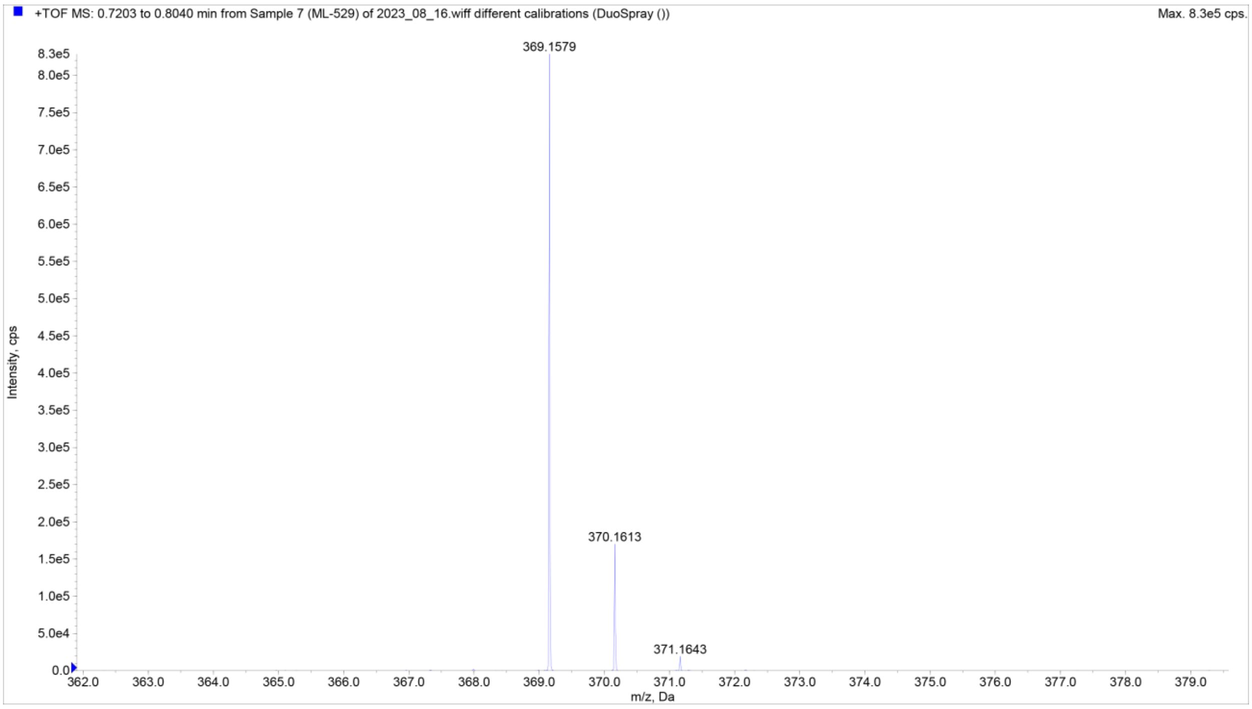

6.25 **ESI-HRMS** spectrum of diaryldihidrophenazinium tosylate **1Phz$^+$OTs$^−$**

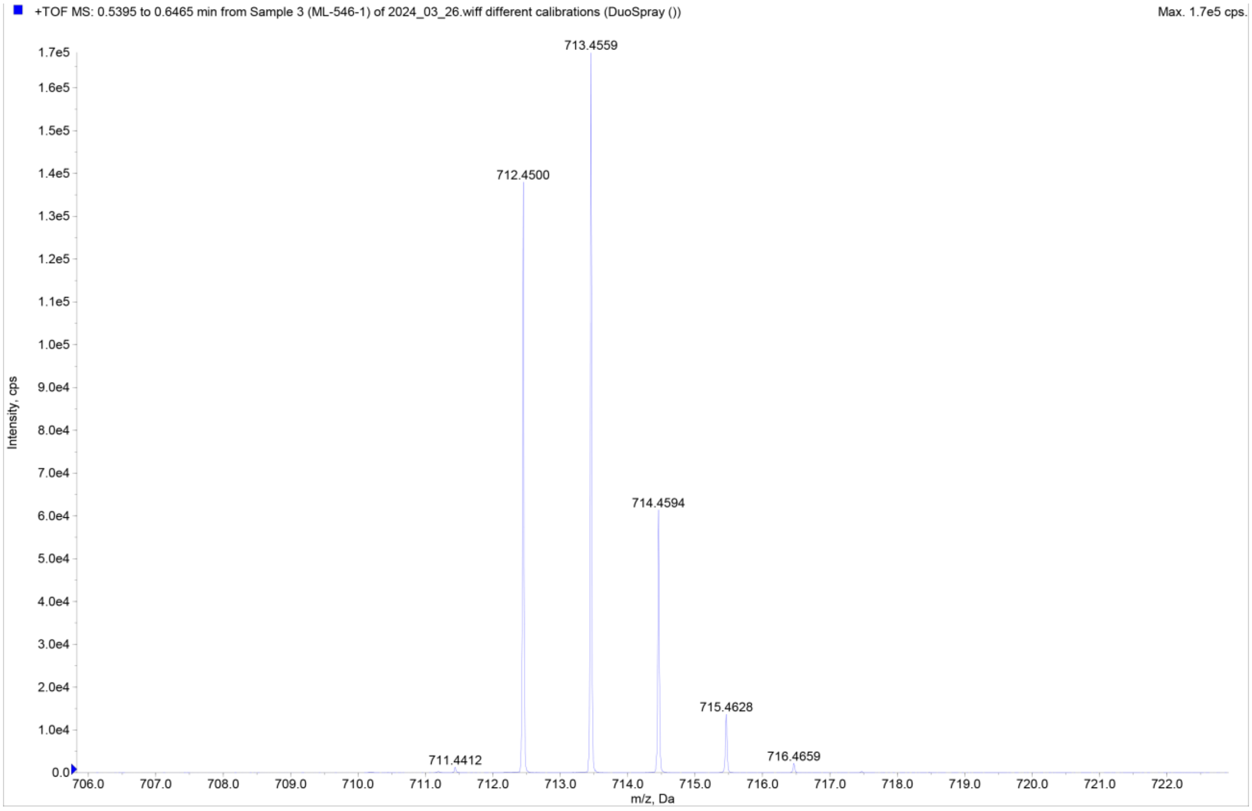

7. CV of bis(trifluoroacetoxy)iodobenzene (PIFA)

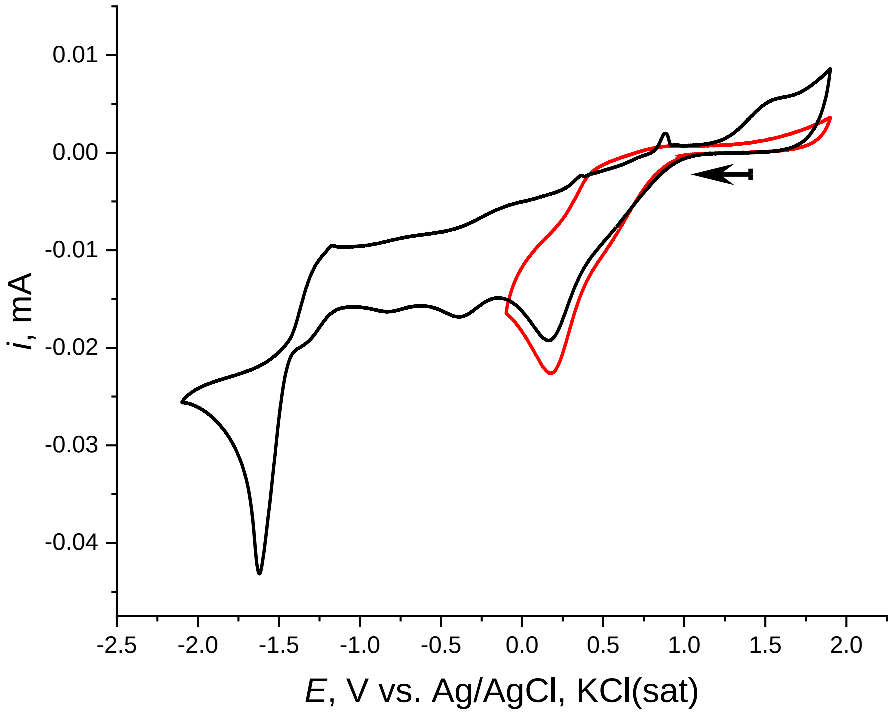


MeCN, 0.1 V/s, Pt, 0.1 M $Bu_4NBF_4$

8. Cyclic voltammpgram of **S1**$^+$**OTs**$^-$

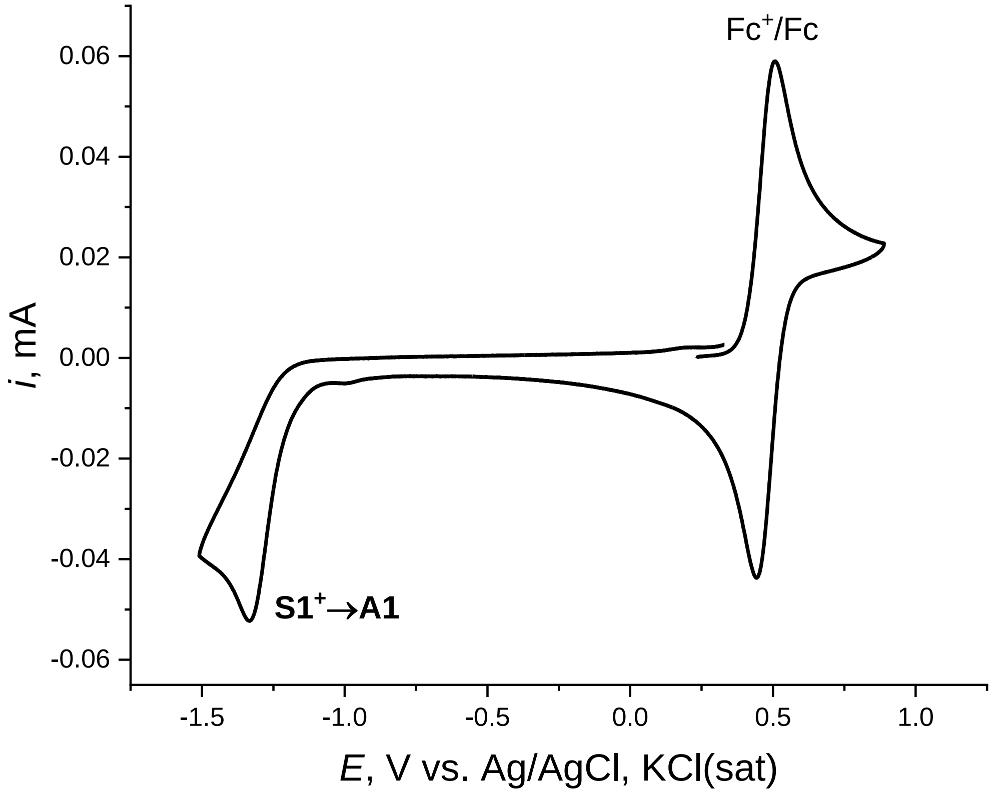


MeCN, 0.1 V/s, GC, 0.1 M $Bu_4NBF_4$
